# Disruption of the *OsWRKY71* transcription factor gene results in early rice seed germination under normal and cold stress conditions

**DOI:** 10.1186/s12870-024-05808-9

**Published:** 2024-11-18

**Authors:** Santiago Bataller, James A. Davis, Lingkun Gu, Sophia Baca, Gaelan Chen, Azeem Majid, Anne J. Villacastin, Dylan Barth, Mira V. Han, Paul J. Rushton, Qingxi J. Shen

**Affiliations:** School of Life Sciences, University of Nevada, 4505 Maryland Parkway, Las Vegas, Las Vegas, NV 89154-4004 USA

**Keywords:** Seed germination, WRKY transcription factors, RNA-sequencing, *Oryza sativa*, Embryos, Cold temperature, Quantitative trait loci (QTL)

## Abstract

**Background:**

Early seed germination in crops can confer a competitive advantage against weeds and reduce the time to maturation and harvest. WRKY transcription factors regulate many aspects of plant development including seed dormancy and germination. Both positive and negative regulators of seed germination have been reported in many plants such as rice and Arabidopsis. Using a transient expression system, we previously demonstrated that OsWRKY71 is a negative regulator of gibberellin (GA) signaling in aleurone cells and likely forms a “repressosome” complex with other transcriptional repressors. Hence, it has the potential to impact seed germination properties.

**Results:**

In this study, we demonstrate that *OsWRKY71*, a Group IIa WRKY gene, appeared at the same time as seed-bearing plants. Rice mutants lacking *OsWRKY71* have seeds and embryos that germinate earlier than wildtype controls. In *oswrky71* aleurone layers, α-amylase activity was hypersensitive to stimulation by GA_3_ and hyposensitive to inhibition by abscisic acid (ABA). Early germination in *oswrky71* intact seeds was also hyposensitive to ABA. Transcriptomic profiling during embryo germination and early post-germination growth demonstrates that OsWRKY71 influences the expression of 9–17% of genes in dry and imbibing embryos. Compared to wildtype embryos, the mutant transcriptomes have large temporal shifts at 4, 8 and 12 h after imbibition (HAI). Importantly, many genes involved in the ABA-dependent inhibition of seed germination were downregulated in *oswrky71-1*. This mutant also displayed altered expression of multiple ABA receptors (*OsPYLs/RCARs*) that control ABA signaling and the VP1-SDR4-DOG1L branch of ABA signaling that promotes seed dormancy. Association studies reveal an *OsWRKY71*-containing quantitative trait locus involved in low-temperature seed germinability, *qLTG-2*. Indeed, *oswrky71* seeds germinated early at 15 °C.

**Conclusions:**

Rice Group-IIa WRKY transcription factor OsWRKY71 is a master regulator of germination that influences the expression of 9–17% of genes in dry and imbibing embryos. It is also most likely the primary candidate of low-temperature seed germinability QTL, *qLTG-2*. We propose that knockouts of *OsWRKY71* can generate rice varieties with improved germination properties under normal or low-temperature conditions.

**Supplementary Information:**

The online version contains supplementary material available at 10.1186/s12870-024-05808-9.

## Background

Seed germination is a pivotal phase in the life cycle of seed plants, which drives the evolution of highly controlled regulatory mechanisms [1–4]. It is the resumption of embryo growth upon rehydration, which covers all events from imbibition to the protrusion of the embryonic axes. The rapid and uniform germination of seeds is important for agriculture and certain industrial processes, such as crop breeding and grain malting. High germination rates at low temperatures (i.e., low-temperature germinability) are also essential for rice varieties adapted to the direct seeding culture [5]. Thus, improving seed germination is at the forefront of achieving food security [6].

The ‘hormone balance theory’ states that the bioactive ABA and GA ratio primarily determines whether a seed remains dormant or germinates [7]. Various environmental factors affect ABA/GA ratio and the embryo’s responsiveness to these phytohormones [3, 8]. ABA sensitivity is affected by several positive regulators of ABA signaling, such as *VP1* [9, 10] and the VP1-activated *Seed Dormancy 4* (*SDR4*) in rice [11]. Later in seed maturation, ABA sensitivity decreases and GA sensitivity increases, which favors germination over dormancy [3]. In this stage, the seed also gradually becomes more sensitive to environmental stimuli [12]. The ‘relief of repression’ model suggests that GA promotes seed germination by degrading a known repressor of GA signaling, the DELLA protein [13–17]. Although the role of ABA and GA in dormancy and germination are widely recognized in genetic and physiological studies, recent findings have also demonstrated the participation of other phytohormones in the regulation of seed dormancy and germination [18–22].

WRKY transcription factors regulate many aspects of plant development including seed dormancy and germination. Arabidopsis WRKY41 directly regulates *ABSCISIC ACID INSENSITIVE 3* (*ABI3*) through promoter binding, with loss-of-function *wrky41* mutants showing reduced *ABI3* expression and reduced sensitivity to ABA during germination and early seedling growth [23]. Three group I Arabidopsis WRKYs, WRKY2, -20 and -44 (TTG2) regulate the expression of *FUSCA3* (*FUS3*), a master regulator of seed maturation and dormancy [24]. Knockout and RNAi lines of *OsWRKY29*, which regulates *ABRE BINDING FACTOR 1* (*OsABF1*) and *VIVIPAROUS 1* (*OsVP1*) genes, had enhanced seed dormancy [25]. Several WRKY genes decrease ABA signaling to promote Arabidopsis seed germination [see 26, for review]. For example, OsWRKY50 directly binds to the promoter and inhibits the expression of the ABA biosynthesis gene *NINE-CIS-EPOXYCAROTENOID DIOXYGENASE 5* (*OsNCED5*). Overexpression of *OsWRKY50* results in seed germination that is hyposensitive to the inhibiting effects of ABA [27].

Using transient expression in barley aleurone layers, we showed that OsWRKY71 negatively regulates a GA-induced α-amylase gene, *HvAmy32b*, by binding to a W-box in its promoter and preventing activation by GA-responsive MYB (GAMYB) [28, 29]. OsWRKY71 is also induced by ABA and repressed by GA [28–30]. Furthermore, OsWRKY71 repression of amylase gene expression is enhanced by interacting with transcriptional regulators, suggesting this repression function is mediated by a “repressosome” complex [29, 31]. Overall, OsWRKY71 appears to be a transcriptional repressor of GA signaling in aleurone cells, potentially impacting seed germination properties. In this study, we demonstrate that rice mutants lacking *OsWRKY71* germinate early due to the altered expression of many genes, including those involved in ABA signaling, mobilization of stored reserves, cell wall loosening, and epiblast tissue weakening. Association studies of OsWRKY71-influenced genes and quantitative trait loci (QTLs) for germination speed (GERMSP) identified an *OsWRKY71*-containing QTL, *qLTG-2*, which is associated with faster seed germination speed at low temperatures [32]. Our results demonstrate that the *oswrky71* early-seed-germination phenotype persists at low temperatures, indicating that *OsWRKY71* is a primary gene of *qLTG-2*. We propose that rice mutants deficient in *OsWRKY71* expression can be used to improve seed germination properties under both normal and cold-stress conditions.

## Results

### Group IIa *WRKYs* co-emerged with seed-bearing plants

We conducted a systematic study on the evolution of the whole *WRKY* gene superfamily across various taxa [33]. In this research, we focused on how *OsWRKY71* and other Group IIa *WRKYs* have evolved from ancestral *WRKY* genes. ​This information may provide insights into linking the evolution of Group II *WRKY* genes with the evolution of seed-bearing plants, particularly since OsWRKY71 plays a crucial role in seed germination (as discussed below). We mapped the emergence of different *WRKY* groups onto a timeline containing representative species from different stages of plant evolution (Supplemental Figure S1A). Group I *WRKYs* are the ancestral type and the only group of *WRKY* genes in unicellular algae. In contrast, Group IIa genes (e.g.,* OsWRKY71*) were the last group to evolve, as they are absent from lycophytes (e.g., spike moss *Selaginella moellendorffii*) but are present in angiosperms. Thus, *OsWRKY71* and other Group IIa *WRKY* genes were late to evolve and their appearance occurred at the same time as the appearance of seeds, seed germination, and more complex defense responses. Evidence shows that Group IIa WRKY transcription factors regulate both seed germination and defense responses [29, 33].

To identify any OsWRKY71 features that might be critical to seed-specific functions, OsWRKY71 orthologs from various species spanning plant evolution were analyzed with CodeML (Supplemental Table S1 and Supplemental Methods S1). This analysis identified 74 amino acids in OsWRKY71 that experienced positive selection relative to the orthologs from 12 pre-selected species and their positions were mapped onto a protein diagram of OsWRKY71 relative to the leucine zipper, WRKYGQK, and C_2_H_2_ motifs (Supplemental Figure S1B). Despite having approximately 21% of the codons in the gene undergoing adaptive evolution, only three sites targeted the DNA-binding WRKY domain and only affected the zinc finger motif. The entire WRKY motif exhibits remarkable conservation, and interestingly, the 27 codon sites directly upstream of this sequence. DNA-binding motifs in transcription factors tend to be highly conserved due to evolutionary pressure, where any significant mutation that alters these sites is likely to be deleterious and detrimental to the organism, leading to the maintenance of this specific sequence throughout evolution. This alludes to the functional importance of OsWRKY71’s DNA-binding motifs.

### Isolation and qRT-PCR analysis of *oswrky71 dSpm* mutants

**Fig. 1:**
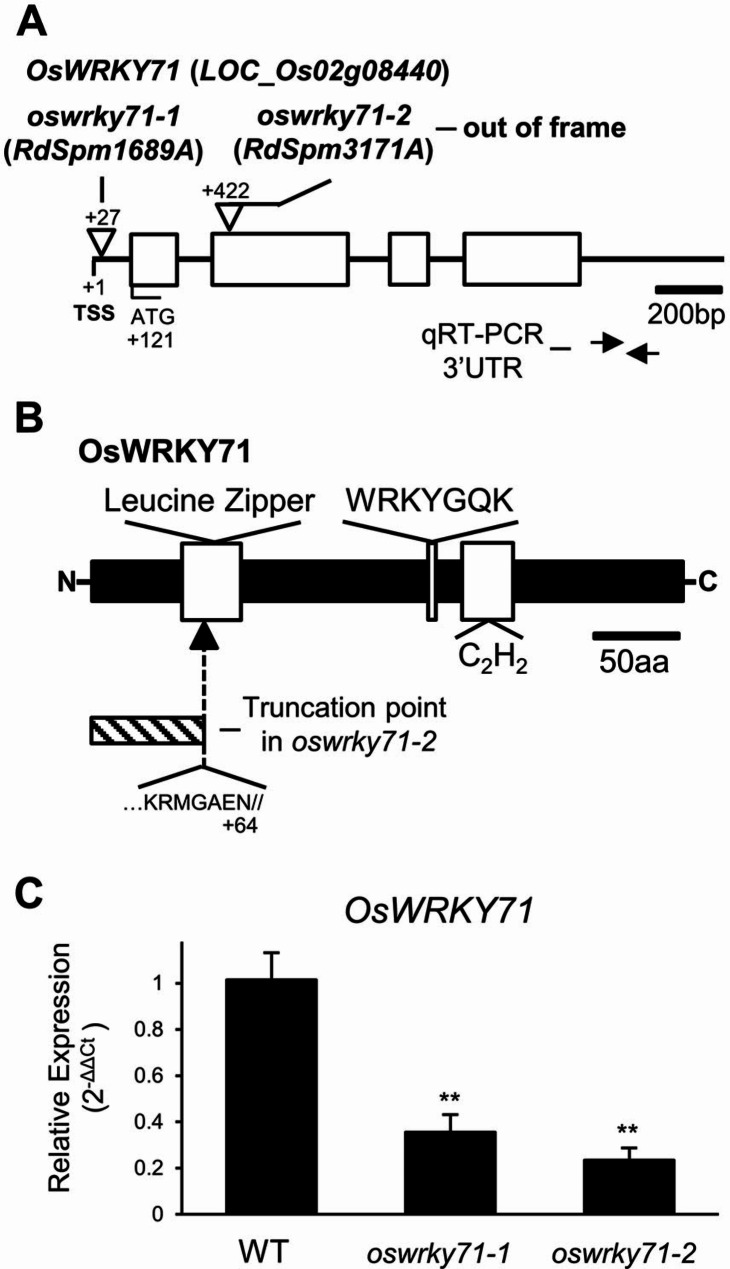
Genetic organization and qRT-PCR verification of *OsWRKY71 dSpm* mutations. **(A)** Gene diagram for *OsWRKY71* showing the position of *dSpm* transposon insertions in *oswrky71-1* and *oswrky71-2*. Transcriptional start site (TSS) and start codon (ATG) are marked. Boxes indicate exons. Triangles with arrows represent the position of each insert. Number of bp downstream from TSS are shown. Free arrows represent the position of primers used in qRT-PCR, which target the 3’UTR of *OsWRKY71*. Scale is 200 bp. **(B)** Protein model for OsWRKY71, with the leucine zipper, WRKYGQK, and C_2_H_2_ domains labeled and marked by white boxes. Scale is 25 aa. **(C)** qRT-PCR quantification of *OsWRKY71* expression in wildtype (WT), *oswrky71-1*, and *oswrky71-2* mutants. *Actin* (*LOC_Os03g50885*) was used as a housekeeping gene. Data represents the mean of three biological replicates ± SEM. Asterisks indicate significant differences determined by Student’s *t*-test (*P* < 0.01)

Two independent rice mutants containing stable *dSpm* transposon disruptions in *OsWRKY71* were obtained from a publicly available collection [34], named *oswrky71-1* (*RdSpm1689A*; 5’UTR insertion) and *oswrky71-2* (*RdSpm3171A*; 2nd exon insertion) (Fig. 1A). DNA sequences around the transposon insertion sites of the mutants are shown in Supplemental Figure S2. On a protein level, *oswrky71-1* is a 5’UTR insertion that does not disrupt the coding sequence, whereas *oswrky71-2* disrupts the leucine zipper motif associated with protein-protein interactions (Fig. 1B). A PCR-based breeding strategy was performed to ensure homozygosity of the *oswrky71* mutations (Supplemental Table S2).

Quantitative reverse transcription-PCR (qRT-PCR) was used to determine whether any residual *OsWRKY71* transcripts were present in each mutant (Supplemental Table S2). RNA was isolated from wildtype and mutant aleurone layers, and qRT-PCR was performed using oligonucleotides targeted to 3’UTR of *OsWRKY71* (Fig. 1A). Transcript abundance was reduced 3-fold in *oswrky71-1* and 4.3-fold in *oswrky71-2* relative to wildtype levels (Fig. 1C). Thus, *oswrky71-1* is a 5’UTR knockdown mutant with residual full-length *OsWRKY71* transcripts, whereas *oswrky71-2* is a knockout mutant with a disrupted protein coding sequence.

### α-Amylase sensitivity to GA and ABA is altered in *oswrky71* half seeds

**Fig. 2:**
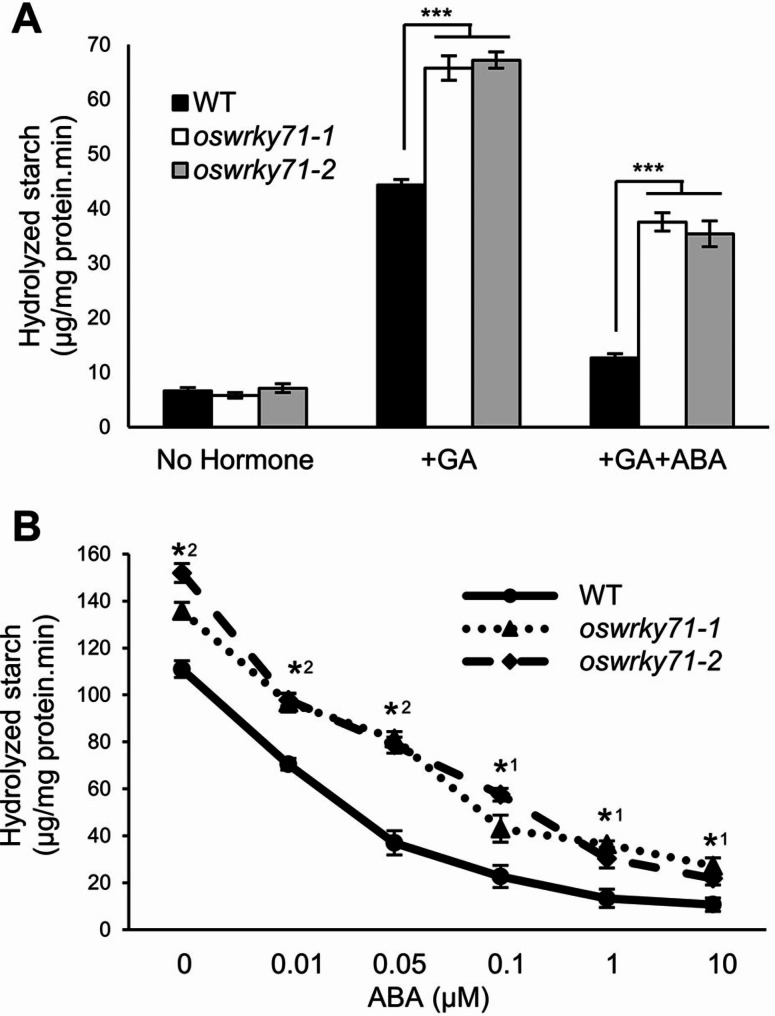
Increased α-amylase activity in the *oswrky71* aleurone cells is GA hypersensitive and ABA hyposensitive. **(A)** α-Amylase activity in wildtype and *oswrky71* mutant half seeds (no embryos) treated for 20 h with no hormone, 5 µM GA_3_, or 5 µM GA_3_ and 0.05 µM ABA. All activity data represents the mean of three biological replicates ± SEM. Asterisks indicate significant differences by Student’s *t*-test (*P* < 0.001). **(B)** Changes in α-amylase activity of wildtype and *oswrky71* mutant half seeds upon treatment with 5 µM GA_3_ and varying ABA concentrations. Asterisks indicate significant differences by the Student’s *t*-test (*P* < 0.05); number beside the asterisk indicates whether the difference occurs in one or both mutant lines

To investigate the effect of *OsWRKY71* mutations on the phytohormone regulation of starch metabolism, α-amylase activity assays were performed on aleurone tissues treated with either GA, GA and ABA, or mock controls. Half seeds were prepared by removing GA-producing embryos and scutellum tissues. When stimulated with just GA, all lines showed strong increases in α-amylase activity, with *oswrky71* mutants being 1.5-fold elevated relative to wildtype (Fig. 2A). While co-treatments with GA and ABA caused activity reductions for all genotypes, *oswrky71* α-amylase activity remained 2.8-fold increased over wildtype controls (Fig. 2A). Compared to the maximal GA-stimulated values for each genotype, ABA treatment caused a 3.5-fold reduction in wildtype, whereas *oswrky71* experienced less severe 1.8-fold reductions. This assay was repeated with co-treatments of GA and varying concentrations of ABA (0–10 µM), with both *oswrky71* mutants displaying elevated α-amylase activity from 0 to 0.05 µM ABA (Fig. 2B). These results suggest that α-amylase activity in *oswrky71* seeds is both hypersensitive to GA induction and hyposensitive to ABA repression.

### *oswrky71* seeds and isolated embryos germinated early

**Fig. 3:**
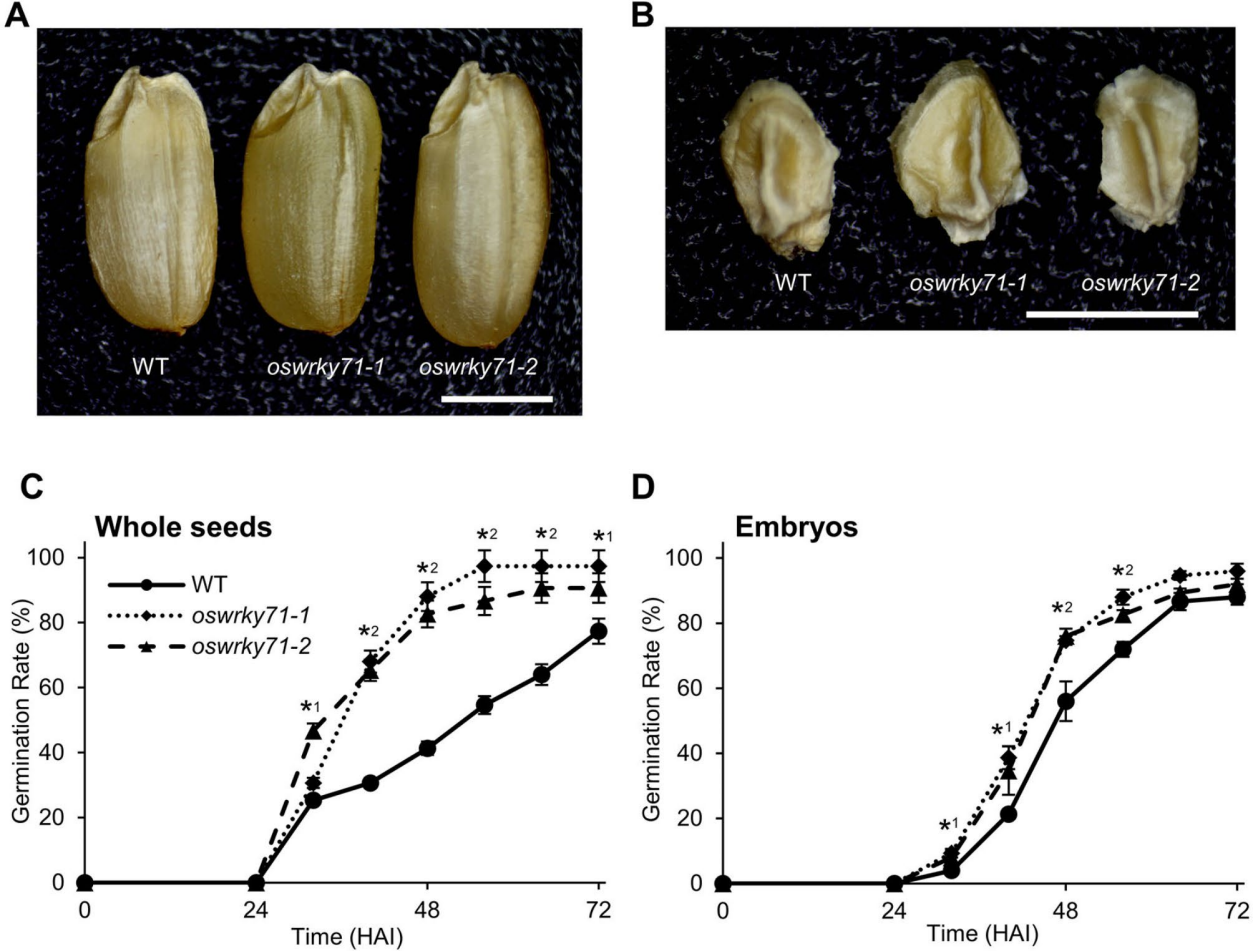
*oswrky71* seeds and isolated embryos germinate early. **(A, B)** Representative images and **(C, D)** quantification of germination percentages for wildtype (WT) and *oswrky71* seeds and isolated embryos. Scale bars are 2 mm. Seeds were incubated in water in the dark and germination rate (%) was measured every 8 h. Seeds were considered germinated when the radicle or the coleoptile is approximately 2 mm. Data represents the mean of three biological replicates ± SEM, with each replicate germination assay containing 25 seeds. Asterisks indicate significant difference relative to wildtype in a Student’s *t-*test (*P* < 0.05); number beside the asterisk indicates whether the difference occurs in one or both mutant lines

To determine whether the loss of OsWRKY71 could affect seed germination percentages, we performed dark-grown germination assays in water. Rice seeds are considered germinated when the emerging root or shoot tissue protrudes from the seed coat [35]. To avoid the difficulties of catching a germinating seed at initial protrusion, which occurs rapidly over a few hours, we relied on an established “germination/early-seedling growth” metric whereby seeds are marked as germinated at 2 mm of radicle or coleoptile growth [36, 37]. Importantly, no obvious morphological differences were observed between the dry seeds or embryos of wildtype and *oswrky71* mutants (Fig. 3A, B and Supplemental Figure S3).

While visibly normal, the percentage of seeds germinated for *oswrky71-2* was higher than wildtype even at 36 HAI. By 48 HAI, the germination percentage for both mutants was 3-fold higher than wildtype and remained higher even at 64 HAI (Fig. 3C). The time to reach a 50% maximal germination (T_50_) was determined using the Farooq method (Farooq et al., 2005), with mutant seeds reaching T_50_ at 35.6 HAI (*oswrky71-1*) and 31.5 HAI (*oswrky71-2*), or 20.5–29.7% earlier than wildtype at 44.8 HAI. Thus, *oswrky71* mutants display an early seed germination phenotype (see Supplemental Movie S1 for time-lapse video).

Starch metabolism in the endosperm is primarily involved in Phase II of germination processes [38], which occurs ~ 4 days after imbibition and is not required for embryo germination [39, 40]. To address whether the *oswrky71* early-seed-germination phenotype is endosperm dependent, we isolated embryos from wildtype and *oswrky71* and repeated our germination assay. Similar to whole seeds, *oswrky71-1* embryos showed increased germination percentages from 32 to 56 HAI and *oswrky71-2* showed increases from 48 to 56 HAI, relative to wildtype (Fig. 3D). At 64 HAI, all lines approached maximum germination. Mutant embryos reached T_50_ at 42.0 HAI (*oswrky71-1*) and 41.9 HAI (*oswrky71-2*), or 7.7–7.9% earlier than wildtype at 45.5 HAI. These results indicate that early germination of *oswrky71* seeds is at least partially attributed to internal changes in the embryo.

### RNA-seq of isolated embryos identified altered gene expression in *oswrky71-1* during germination

**Fig. 4:**
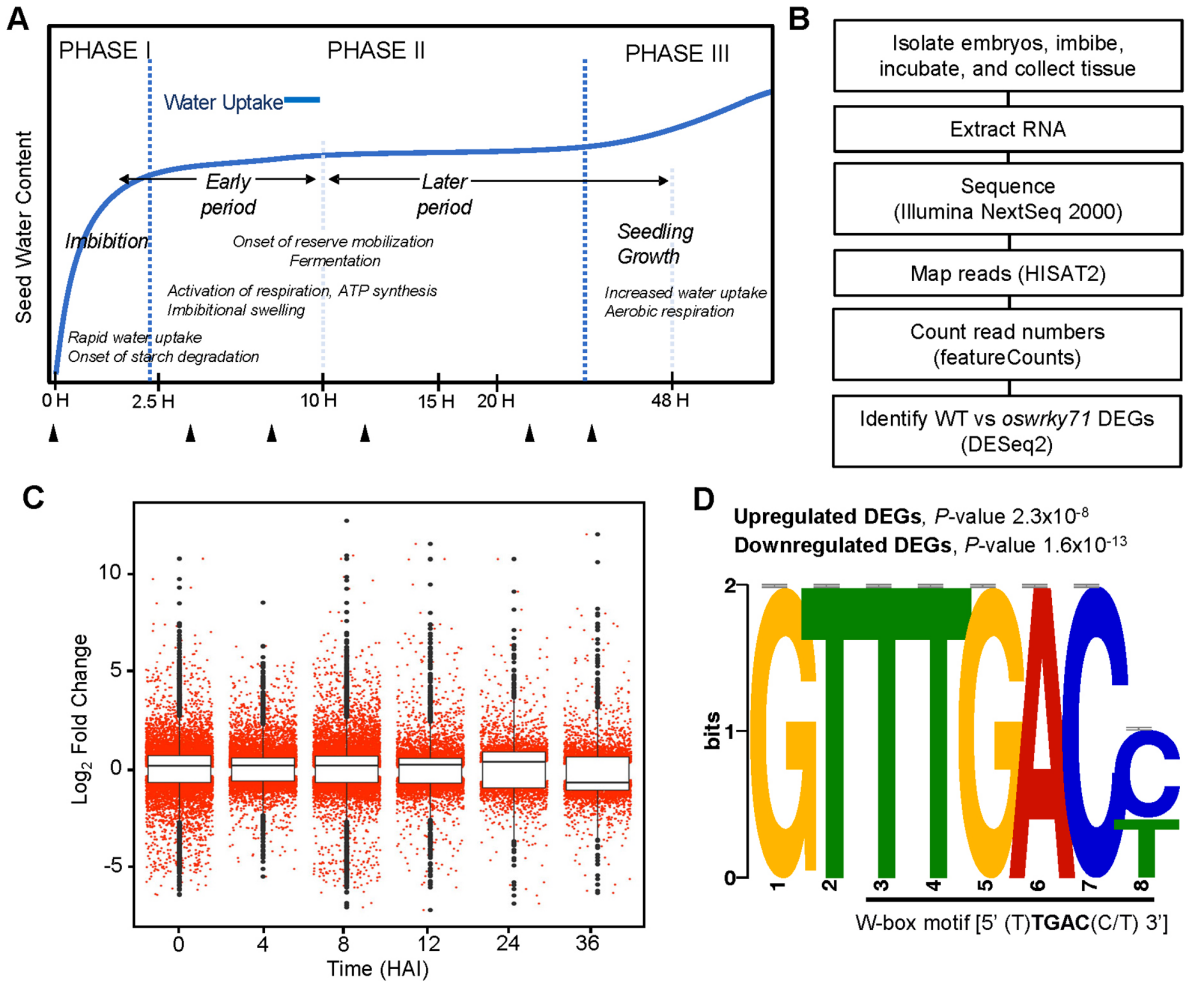
Dynamic transcriptomic changes in *oswrky71-1* embryos during germination. **(A)** Schematic representation of key processes during rice seed germination and the onset of post-germination growth. Time points for RNA-seq analysis are marked by black triangles on the x-axis (0-hr, 4-hr, 8-hr, 12-hr, 24-hr, 36-hr). **(B)** Flowchart showing pathway for RNA-seq and subsequent bioinformatic analyses. **(C)** Expression profile of DEGs along the time-course. Y-axis values represent log_2_ fold change in the mutant relative to wildtype control. Box plot components are as follows: box edges are first and third quartiles, central line is the median, whiskers are the upper and lower 1.5x interquartile ranges; red dots, individual genes; black dots, outliers. **(D)** Identified W-box motif enriched in *oswrky71-1* DEGs at 8 HAI, when the greatest number of DEGs were detected

To investigate the transcriptional changes associated with early seed germination in *oswrky71*, an RNA-seq analysis was performed on isolated embryos during a time course spanning the early phases of germination and ending with the onset of post-germination processes (Fig. 4A, see Supplemental Table S3 for mapping frequencies). Isolated embryos were used as a minimalist tissue system that displayed early *oswrky71* germination (Fig. 3D), as it is easy to prepare and process, and avoids rapid wounding responses from mechanical dissection post-imbibition. Wildtype and os*wrky71-1* embryos were compared at the following time points: 0 h (i.e., dry embryo), 4, 8, 12, 24, and 36 HAI (Fig. 4A, B).

Comparing wildtype and *oswrky71*, we identified 9,537 differentially expressed genes (DEGs, padj ≤ 0.05, Likelihood ratio test) out of 55,987 unique MSU7 gene models, or 17% of all genes at 0 HAI (5,054 up, 4,483 down), 14% at 4 HAI (4,288 up, 3,479 down), 17% at 8 HAI (5,259 up, 4,491 down), 10% at 12 HAI (3,120 up, 2,277 down), 10% at 24 HAI (2,650 up, 2,689 down), and 9% at 36 HAI (2,304 up, 2,658 down) (Fig. 4C, Supplemental Figure S4A). Counts normalized using the median of ratios method in DESeq2 for DEGs at one or more timepoints are shown in Supplemental Table S4. Upregulated and downregulated DEG lists were cross-referenced to find DEGs that occurred at more than one time point, and the number of DEGs occurring in each of the 63 potential combinations was displayed in an UpSet plot (Supplemental Figure S4B, C). Of these, a total of 107 upregulated and 59 downregulated DEGs were common to all time points (Supplemental Table S5A). The largest common DEGs were shared between 4 and 8 HAI for up-regulated genes (749 genes), and among 0, 4, and 8 HAI for down-regulated genes (667 genes). As a confirmation of the high quality of our dataset, *OsWRKY71* was identified as downregulated at every time point.

To identify enriched motifs from the *cis*-regulatory elements in the promoters of differentially expressed genes identified by RNA-seq, we performed an ab initio analysis using DREME [41]. Promoter sequences located 2 kb upstream of the transcription start site for the top 1,000 upregulated and downregulated *oswrky71* DEGs were analyzed and ranked by log_2_FC values. A motif [GTTTGAC(C/T)] was identified as a highly enriched *cis-*acting element, which occurred in 32% (upregulated) and 40% (downregulated) of the analyzed promoters (Fig. 4D). Importantly, this element contains a canonical W-box [(T)TGAC(C/T)] sequence known to be the binding site of WRKY transcription factors. This suggests that DEGs containing these W-boxes might be directly regulated by OsWRKY71 or influenced by other WRKYs that are themselves regulated by OsWRKY71.

### *oswrky71-1* has reduced expression of important ABA signaling genes

To investigate the transcriptional changes associated with early seed germination in *oswrky71*, an RNA-seq analysis was performed on isolated embryos during a time course spanning the early phases of germination and ending with the onset of post-germination processes (Fig. 4A, see Supplemental Table S3 for mapping frequencies). Wildtype and os*wrky71-1* embryos were compared at the following time points: 0 h (i.e., dry embryo), 4, 8, 12, 24, and 36 HAI (Fig. 4A, B).

**Fig. 5:**
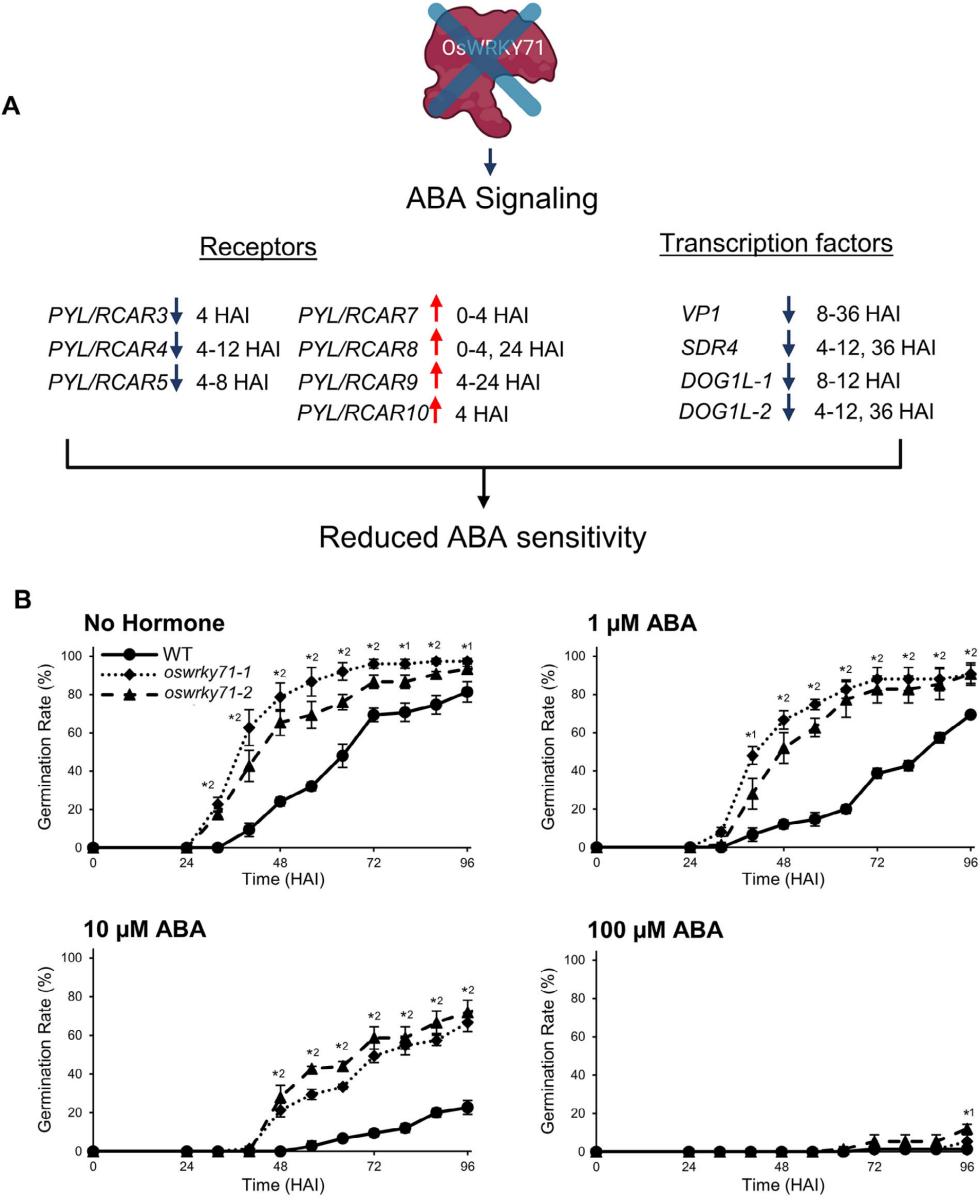
*oswrky71* early seed germination is hyposensitive to ABA treatment. **(A)** Model showing the reduced expression of certain ABA signaling genes in *oswrky71* embryos during germination. Downward blue arrows indicate reduced expression in *oswrky71* embryos. Numbers indicate the RNA-seq time points in hours after imbibition (HAI) when the genes were differentially expressed. **(B)** Quantification of germination percentages for wildtype (WT), *oswrky71-1*, and *oswrky71-2*. Whole seeds were incubated at 28 °C in the dark on agar plates enriched with ½x MS growth media (0.05% MES (w/v), pH = 5.8) and supplemented with either 0 µM ABA (No Hormone), 1 µM ABA, 10 µM ABA, or 100 µM ABA. Germination was defined as a radicle or coleoptile reaching approximately 2 mm in length. Germination was tracked for an extended period due to the germination inhibiting effects of ABA. Data represents the mean of three biological replicates ± SEM, with each replicate germination assay containing 25 seeds. Asterisks indicate significant differences relative to wildtype in Student’s *t-*test (*P* < 0.05); number beside the asterisk indicates whether the difference occurs in one or both mutant lines

Many ABA signaling genes showed altered expression levels in *oswrky71-1* embryos (Supplemental Table S5B). For the family of *Pyrabactin resistance-like/Regulatory Component of ABA Receptors* (*PYL/RCARs*) that promote ABA signaling, post-imbibition reductions were detected for the highest expressed members: *PYL/RCAR3* (4 HAI), *PYL/RCAR4* (4–12 HAI), and *PYL/RCAR5* (4 and 8 HAI). PYL/RCAR7 is known to be a potent repressor of PP2C activity. In vitro [42] and transgenic overexpression of PYL/RCAR5 has been shown to delay rice germination [43]. In contrast, more lowly expressed members showed induction: *PYL/RCAR7* (0 and 4 HAI), *PYL/RCAR8* (0, 4, 24 HAI), *PYL/RCAR9* (4–24 HAI) and *PYL/RCAR10* (4 HAI) (Fig. 5A and Supplemental Table S5B). As PYL/RCARs promote ABA signaling, the observed net expression reduction in some *PYL/RCARs* in *oswrky71-1* is predicted to reduce sensitivity to ABA and increase germination speeds (Fig. 5). How those receptors with enhanced expression contribute to the reduced ABA sensitivity of the *oswrky71-1* mutant seeds remains to be studied.

Dynamic regulation was detected for the critical ABA signaling and seed maturation and germination regulator *VP1* [11, 44], which was upregulated at 0 HAI but was downregulated at 8 and 36 HAI (Supplemental Table S5B). In support of the post-imbibition reductions in *VP1*, we also detected post-imbibition reductions in the VP1-induced gene *Seed Dormancy 4* (*SDR4*; 4–12, 36 HAI). Similarly, *oswrky71* showed post-imbibition reductions for SDR4-induced *Delay of Germination1-like*s (*DOG1Ls*), which positively regulate ABA signaling by reducing the PPase activity of ABA-receptor complexes [11, 45]. Specifically, *DOG1L-1* was downregulated at 4–12 HAI, *DOG1L-2* at 8–12 and 36 HAI, and *DOG1L-3* at 4–12 and 36 HAI. Together, this data suggests that *oswrky71* embryos display post-imbibition reductions in genes encoding the VP1-SDR4-DOG1L branch of ABA signaling, which could accelerate germination.

### Early seed germination in *oswrky71* is ABA hyposensitive

The altered expression of ABA signaling genes in *oswrky71* embryos suggests the potential for reduced sensitivity to ABA during germination. To test this hypothesis, we performed germination assays with whole seeds on solid agar plates enriched with plant growth media and supplemented with varying concentrations of ABA. Germination was measured for an extended period to account for the germination-delaying effects of ABA. Like previous results, *oswrky71-1* mutants showed increased germination percentages from 32 to 88 HAI on solvent-control plates, whereas *oswrky71-2* was increased from 32 to 72 HAI and at 88 HAI (Fig. 5). Compared to the wildtype T_50_ of 60.9 HAI, *oswrky71-1* and *oswrky71-2* mutants showed 37.9 HAI and 41.7 HAI, respectively (i.e., 37.8–46.2% earlier).

On ABA-supplemented plates, the *oswrky71* early-seed-germination phenotype persisted up to 10 µM ABA. On 1 µM ABA, *oswrky71-1* showed increased germination from 40 to 96 HAI and *oswrky71-2* from 48 to 96 HAI. This resulted in a wildtype T_50_ of 70.6 HAI, whereas *oswrky71-1* and *oswrky71-2* showed 39.7 and 45.9 HAI, respectively (i.e., 35–43.8% earlier). At 10 µM ABA, both mutants showed elevated germination percentages from 48 to 96 HAI, with the T_50_ of *oswrky71-1* (60.2 HAI) and *oswrky71-2* (51.4 HAI) being 18.6–30.6% earlier than the wildtype (74 HAI). At this concentration, the final germination rate of the mutants was over 66%, whereas wildtype showed only 23%. However, all lines showed severely impaired germination at 100 µM ABA. Together, these results indicated that the *oswrky71* early seed germination phenotype is ABA hyposensitive.

### OsWRKY71 functions upstream of other transcription factors in the signaling web that regulates germination

**Fig. 6:**
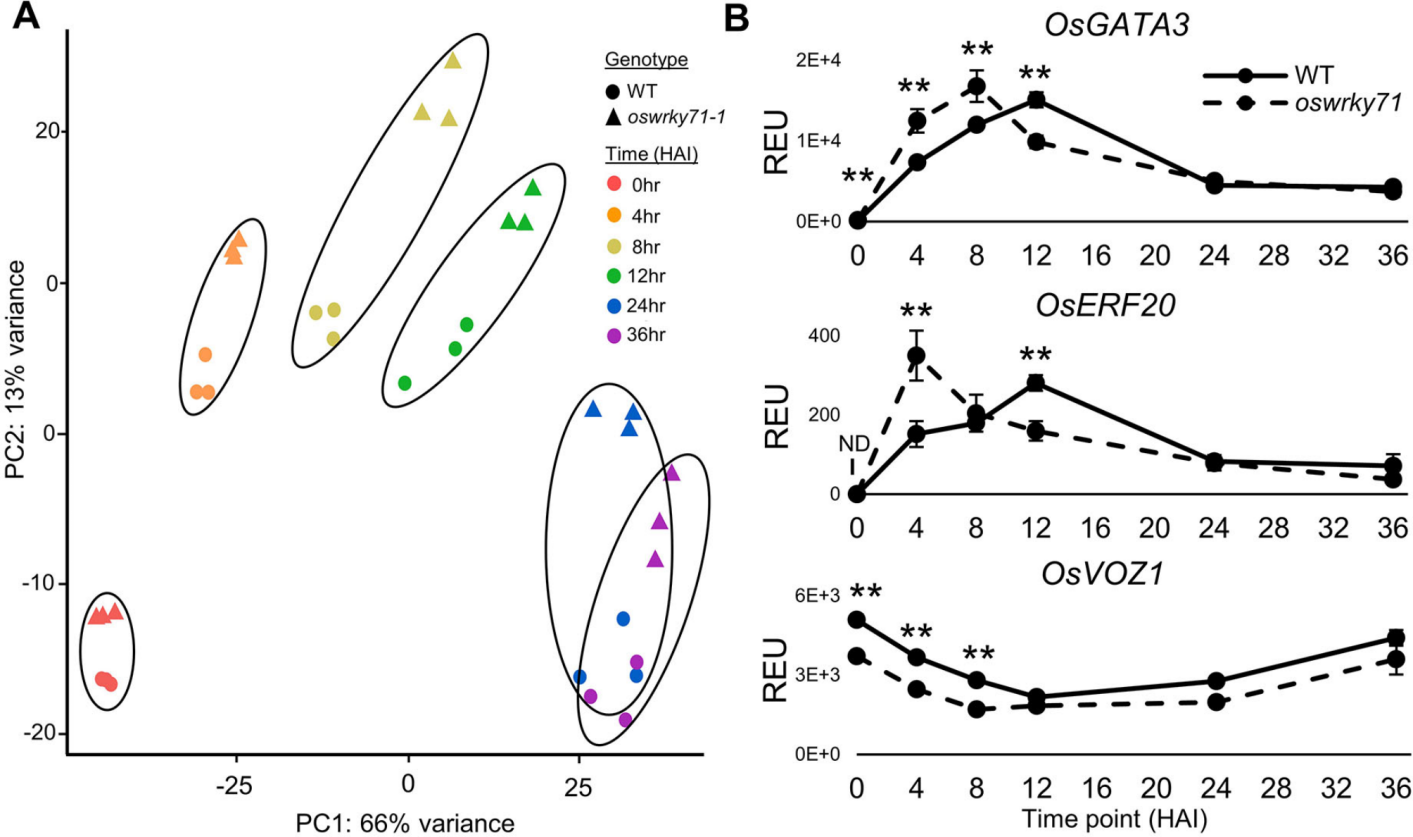
Identification of genes displaying early expression patterns in *oswrky71* embryos. **(A)** PCA plot showing divergence between wildtype (WT) and *oswrky71-1* expression profiles over time. **(B)** Representative genes with early expression patterns compared to the wildtype. Data represents the mean of three biological replicates ± SEM. Asterisks indicate significant difference relative to wildtype (*Padj* < 0.01). REU: Relative Expression Unit, equivalent to the number of mapped reads normalized by the median of ratios method in DESeq2

Our data clearly define and illustrate the germination phenotype of *oswrky71*. It is clear that ABA plays a significant role in the processes that OsWRKY71 regulates in the seed, but what other components contribute to the phenotype? To further investigate, we performed principal component analysis (PCA) analysis on the RNA-seq data from the mutant and wildtype during germination. We identified the 4, 8, and 12 HAI time points as having the largest differences between wildtype and *oswrky71* (Fig. 6A). Thus, a 4-hour cross-lag correlation analysis was performed on these time points to identify genes that show early expression patterns in germinating *oswrky71* embryos. This revealed a total of 103 candidates. Another approach identified DEGs specific to 4, 8 and 12 HAI in wildtype by comparing the transcriptome at one time point to that of each of the remaining time points. We then further narrowed the list to genes encoding transcription factors expressed earlier in the mutant than in wildtype, resulting in a total of 707 genes. One of these genes is *GATA transcription factor 3* (*OsGATA3*, LOC_Os02g56250), which is induced by ABA and hence implicated in ABA signaling [46] (Fig. 6). OsGATA3 is one of 9 candidate rice orthologs of Arabidopsis AtGATA12, a transcription factor that promotes seed dormancy [47]. However, of the putative rice orthologs of *AtGATA12*, only *OsGATA3* is highly expressed during embryo germination and differentially expressed in *oswrky71* (Supplemental Table S5B). In wildtype, *OsGATA3* expression begins low in dry embryos and increases in expression up to 12 HAI, after which expression begins to decline (Fig. 6B). In contrast, in the *oswrky71* mutant, *OsGATA3* expression peaked four hours earlier at 8 HAI and had declined significantly by 12 HAI. The earlier decline in *OsGATA3* transcript levels may reflect an earlier release of dormancy and/or a more rapid onset and speed of germination. This is consistent with the germination phenotype of *oswrky71*.

Ethylene Response Factor 20 (*OsERF20*, LOC_Os02g45420) has been implicated in playing roles in ethylene signal transduction during germination and early seedling growth [48]. No *OsERF20* gene expression was detected in dry embryos for either genotype. Subsequently, in WT, *OsERF20* expression was induced from 4 to 12 HAI, then was reduced afterward (Fig. 6B). Strikingly, in *oswrky71*, expression of *OsERF20* peaked a full eight hours earlier at 4 HAI and then steadily declined. This implicates not only OsERF20 in regulating seed germination and early seedling growth in rice, but also OsWRKY71 in the direct or indirect regulation of *OsERF20*.

The final early-expressed gene is of particular interest because its mRNA level is reduced in the *oswrky71* mutant at all studied timepoints during germination compared to wildtype. The gene is *Vascular plant One-Zinc finger 1* (*OsVOZ1*, LOC_Os01g54930), which encodes a transcription factor involved in plant growth and pathogen defense [49] (Fig. 6B). Overall, these three transcription factor genes illustrate parts of the signaling network that operate during rice seed germination. OsWRKY71 is clearly a crucial upstream component of this network, as judged by its germination phenotype when down-regulated or knocked out and the presence of other transcription factor genes downstream of OsWRKY71.

### *oswrky71* displays altered expression of genes for seed storage mobilization, water transport, and epiblast tissue weakening

Because the phenotype of *oswrky71* shows that OsWRKY71 regulates seed germination, we investigated whether genes involved in well-known germination processes (e.g., seed reserve mobilization, cell wall remodeling genes, etc.) are perturbed in the mutant. Gene ontology (GO) enrichment analyses revealed that two GO terms, “carbohydrate metabolic process” and “hydrolase activity, hydrolyzing O-glycosyl compounds,” were highly enriched in upregulated DEGs (Supplemental Table S6). This suggests that early germination in *oswrky71* embryos is associated with increased expression of genes involved in carbohydrate metabolism. To determine whether this included alterations to starch metabolism, the expression levels of starch metabolism genes were analyzed, including α-amylases (*RAmys*), β-amylases (*BMYs*), α-glucosidases (*ONGs*; i.e., maltases), and debranching enzymes Isoamylases (*ISAs*). Heat map expression profiles of these genes can be found in Supplemental Table S5 (Supplemental Table S5C).

Of the 10 *RAmys* analyzed, two were upregulated (*RAmy1A*, *RAmy5A*) and 4 were downregulated (*RAmy2A*, *RAmy3B*, *RAmy3E*, *RAmy3D*) in dry *oswrky71* embryos (Supplemental Figure S5A, Supplemental Table S5C). By 4 HAI, most α-amylase genes were upregulated (*RAmy1A*, *RAmy1C*, *RAmy3A*, *RAmy5A*, *RAmy3D*, *RAmy3E*). This earlier upregulation of α-amylase genes in the mutant suggests a more rapid breakdown of seed reserves occurs in *oswrky71* compared to wildtype. This is one of the causes leading to the faster germination phenotype. By 8 HAI, only *RAmy1A* and *RAmy5A* were still upregulated, and after 24 HAI, *RAmys* were primarily downregulated (*RAmy3A*,* RAmy3D*, *RAmy3F*).

*BMY* expression in *oswrky71* was upregulated in dry embryos and downregulated post-imbibition. Interestingly, expression of the *ONG* family of maltases was higher in the mutant compared to wildtype, with *ONG1* upregulated from 0 to 12 HAI and 36 HAI, *ONG2/3* being upregulated from 4 to 36 HAI, and *ONG4* being upregulated at 0 and 8 HAI (Supplemental Figure S5A, Supplemental Table S5C). The consistent upregulation of *ONG* maltases suggests that OsWRKY71 is a negative regulator of these genes in embryos. In contrast to other starch-metabolism genes, few changes were detected for *Isoamylases* (Supplemental Table S5C).

To investigate whether sugar transport is also altered in *oswrky71*, we analyzed the expression of two gene families: *Sugar Will Eventually be Exported Transporters* (*SWEETs*) and *Sucrose Transporters* (*SUTs*) (Supplemental Figure S5B, Supplemental Table S5C) [50, 51]. *SWEET1b* and *XA41* (*SWEET14*) were highly upregulated in *oswrky71*. Other upregulated *SWEET*s were *SWEET1a*,* SWEET1b*, *SWEET2a*, and *SWEET4*, which are all implicated in glucose transport [52, 53]. Of the five rice *SUTs*, *oswrky71* showed increased expression of *SUT2*,* SUT4*, and *SUT1*. These genes showed different kinetics, but all showed increased expression levels in the mutant at the early 4 HAI timepoint. *SUT1* is highly expressed in rice seeds and mutants deficient in *SUT1* show impaired germination [54, 55]. Interestingly, *DOF11* (also called *DOF7*, LOC_Os02g47810/Os02g0707200), which directly regulates *SUT1*, *SWEET11*, and *SWEET14* [56], was also upregulated over 2-fold in the mutant at 0–4 HAI and downregulated at later stages (Supplemental Table S5C). Our data, therefore, suggests that OsWRKY71 is upstream of DOF11, either directly or indirectly, causing *oswrky71* mutants to display increased *DOF11* and sucrose-transporter expression at early germination stages. Alternatively, OsWRKY71 could also repress the transporter genes directly as well as through *DOF11*. Thus, the early seed germination phenotype of *oswrky71* is associated with the increased expression of carbohydrate metabolism and sugar transport genes, which could accelerate stored reserve mobilization to the germinating embryo.

Also among the upregulated DEGs in *oswrky71* are genes that encode glutelin seed storage proteins (SSPs) (Supplemental Figure S5C, Supplemental Table S5D), which are metabolized during rice germination to feed embryo growth [57]. Among the 15 glutelin genes, 13 were upregulated > 4-fold in dry embryos and most were upregulated from 0 to 12 HAI. Certain protein metabolism genes were also upregulated in *oswrky71*, including *REP-*1 and other *Cysteine Protease* (*OsCPs*) genes that are involved in SSP degradation and amino acid mobilization (Supplemental Figure S5D, Supplemental Table S5D) [58], multiple *Serine Carboxypeptidases* (*OsSCPs*) that might also metabolize SSPs, and four amino acid transporters *AAP1*, *AAP3*, *AAP6*, and *AAP11A* (Supplemental Figure S5E).

Finally, we analyzed the expression of genes involved in cell wall remodeling (Supplemental Figure S5F, Supplemental Table S5E) and water uptake (Supplemental Table S5F). Cell wall remodeling gene families analyzed here included: *Expansins* (*EXPAs*), *Xyloglucan endotransglucosylase/Hydrolases* (*XTHs*), and endo-(1,4)-β-D-glucanases (i.e., cellulases) named *Glycosyl Hydrolase 9s* (*GH9s*) [59]. Water uptake genes were limited to aquaporins, including the *NOD26-like Intrinsic Proteins* (*NIPs*), *Plasma membrane Intrinsic Proteins* (*PIPs*), *Tonoplast Intrinsic Proteins* (*TIPs*), and the *Small and Basic Intrinsic Proteins* (*SIPs*). An interesting highlight of these expression patterns was the upregulation of multiple *EXPAs* in dry *oswrky71* embryos, which could accelerate germination by increasing the rate of cell wall loosening upon imbibition.

### *OsWRKY71* and *oswrky71* DEGs were linked to QTLs associated with low-temperature germinability

QTL studies have facilitated the identification of genes underlying quantitative traits, with thousands of rice QTLs having been grouped and curated by Gramene (https://archive.gramene.org/qtl/) [60]. To identify QTLs linking OsWRKY71 to the regulation of seed germination, we analyzed whether genes differentially expressed (DE) in *oswrky71* were enriched in the germination speed (GERMSP) category of QTLs. GERMSP includes 20 unique QTL entries that are mapped to genomic regions and contain at least one MSU7 gene model. The number of genes that were DE or non-DE in each GERMSP QTL and at each time point are shown in Supplemental Table S7A. QTLs were defined as enriched with DE genes (i.e., enriched QTL) if the odds ratio was above 1 (log adds ratio above 0) and had a *P*-value ≤ 0.05 by Fisher’s Exact Test. Enriched QTLs were found in most time points (0–24 HAI) and included 9 out of 20 GERMSP QTLs (Supplemental Figure S6; Supplemental Table S7B). Four of these enriched QTLs (*AQF121*, *AQF122*, *AQFE110*, and *CQY1*) were enriched throughout 0–24 HAI and are highlighted in bold in Supplemental Table S7B.

One GERMSP QTL enriched from 0 to 24 HAI was *CQY1*, a QTL associated with improved low-temperature germinability and named *qLTG-2* [32]. Importantly, *OsWRKY71* is one of 245 gene models in the *qLTG-2* interval (Supplemental Table S7C). Four genes in *qLTG-2* were commonly DE in *oswrky71-1* embryos at all time points (0–36 HAI), and not surprisingly *OsWRKY71* was one of them. Whether the other three genes participate in OsWRKY71-dependent regulation of seed germination speed warrants further study.

**Fig. 7:**
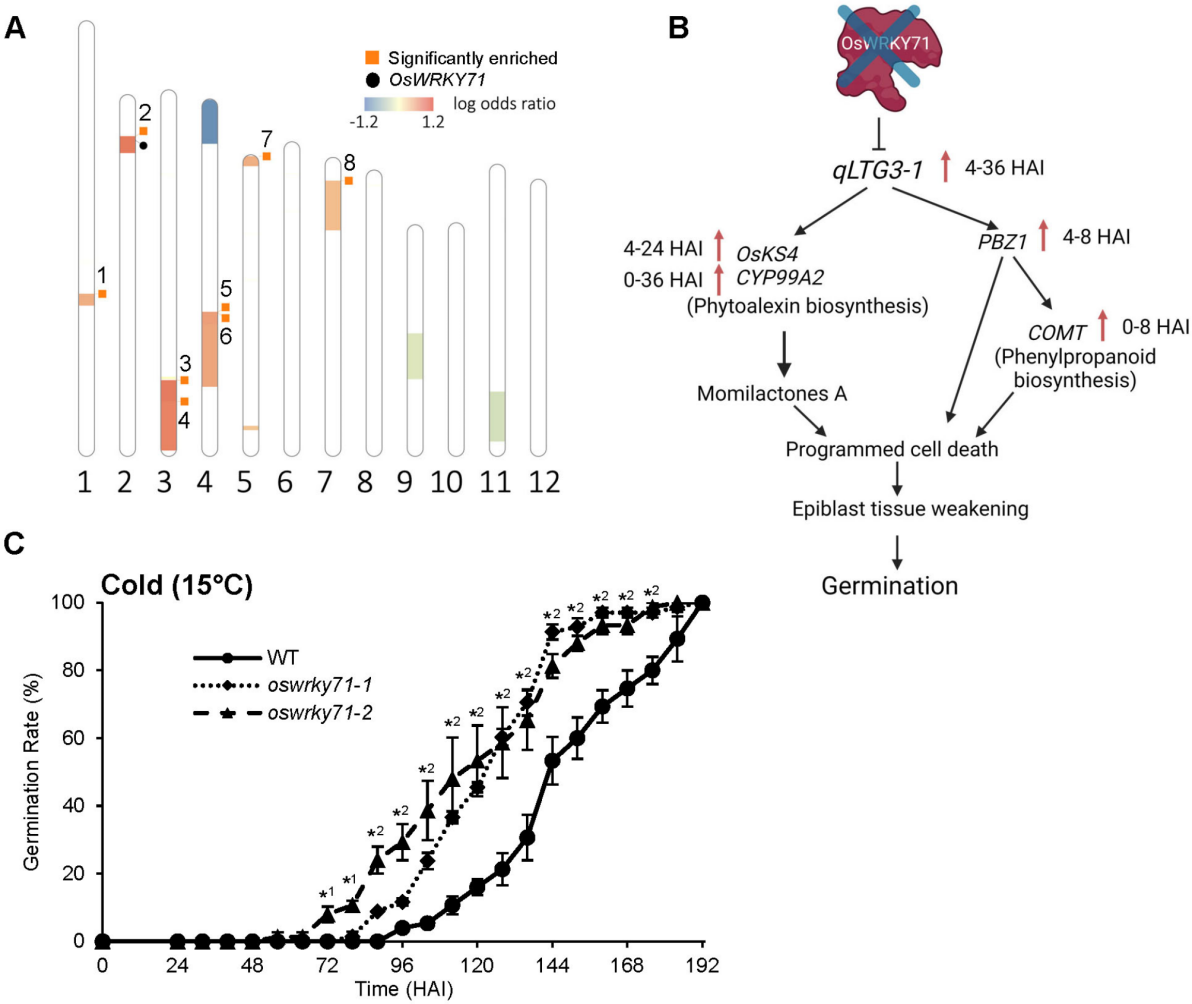
*oswrky71* seeds show improved low temperature germinability. **(A)** Rice chromosomes with regions color-coded by association mapping. Location of GERMSP QTLs on chromosomes are marked with different colors based on the log odds ratios, which resulted from the Fisher Exact Test on if a QTL is enriched with *oswrky71* DEGs. Significantly enriched QTLs with odds ratios above 1.0 (log odds ratios above 0) and p values ≤ 0.05 are marked with orange squares. *OsWRKY71* position is marked with a black dot. The numbers around orange squares represent the Gramene accession numbers of the enriched QTLs: (1) AQF118 [79]; (2) CQY1 [32]; (3) AQF121 [79]; (4) AQF122 [79]; (5) CQY3 [32]; (6) AQFE110 [80]; (7) CQY4 [32]; (8) AQAC008 [81]. **(B)** Chart showing expression changes of *qLTG3-1*, a major QTL controlling low-temperature tolerance at the germination stage, and its target genes in *oswrky71* embryos. Upward red arrows indicate increased expression in *oswrky71* embryos. Numbers indicate the RNA-seq time points in HAI when the genes were differentially expressed. **(C)** Quantification of germination percentages for wildtype (WT) and *oswrky71* mutants in cold conditions. Whole seeds were incubated at 15 °C in the dark on agar plates enriched with ½x MS growth media (0.05% MES (w/v), pH = 5.8). Germination was defined as a radicle or coleoptile reaching approximately 2 mm in length. Germination was tracked for an extended period due to the germination delaying effects of low temperatures. Data represents the mean of three biological replicates ± SEM, with each replicate germination assay containing 25 seeds. Asterisks indicate significant differences relative to wildtype in Student’s *t-*test (*P* < 0.05); number beside the asterisk indicates whether the difference occurs in one or both mutant lines

An additional QTL involved in low-temperature germinability, *qLTG-3-1* (*AQF122*), was also enriched in *oswrky71-1* from 0 to 24 HAI (Fig. 7) [61]. The functionality of this QTL is attributed to a gene encoding a glycine-rich protein, which was upregulated in *oswrky71* from 4 to 36 HAI. *qLTG-3-1* enhances germination under low-temperature conditions by inducing the expression of *Probenazole1-induced* 1 (*PBZ1*), which causes vacuolation and weakening of the epiblast tissue that covers the embryo [62]. Interestingly, *PBZ1* and other targets of *qLTG-3-1* showed increased expression in *oswrky71-1* (Supplemental Figure S5G). For example, *PBZ1* was upregulated > 4-fold in dry embryos and at 8 HAI, whereas a *caffeic acid 3-O-methyltransferase* named *COMT* was upregulated > 2-fold in dry embryos and at 8–12 HAI. *KS4* and *CYP99A2* genes involved in phytoalexin biosynthesis were also highly upregulated with > 4-fold induction at 4–24 HAI and 0–36 HAI, respectively.

A final low-temperature germinability QTL showing differential expression was *qLTG-5* (*CQY4*), a UDP-glucosyltransferase gene (*LTG5*) upregulated 2-fold in *oswrky71* dry embryos. Notably, overexpression of *LTG5* is known to improve low-temperature germinability [63]. Together, these results suggested that OsWRKY71 plays a role in regulating rice seed germination at low temperatures, potentially as the primary gene of *qLTG-2* and acting through the altered expression of multiple other low-temperature germinability QTLs (e.g., *qLTG-3-1*, *qLTG-5*).

### *oswrky71* seeds displayed improved low-temperature germinability

Seed germination assays were repeated at low temperatures (15°C) to determine whether *OsWRKY71* is a primary gene of *qLTG-2*. Assays were performed using whole seeds on solid agar plates supplemented with plant growth media. Seed germination percentages were measured as previously described, but the assay was monitored for an entire week to accommodate the delayed seed germination associated with low temperatures. No wildtype seeds germinated until 4 days after imbibition. In contrast, *oswrky71-1* mutants began germination at 72 HAI and showed elevated germination over wildtype until 176 HAI (Fig. 7C). Compared to the wildtype T_50_ of 145 HAI, *oswrky71-1* and *oswrky71-2* mutants showed 123 HAI and 119 HAI, respectively (i.e., a day earlier). Excitingly, the improved low-temperature germinability of *oswrky71* seeds confirmed that *OsWRKY71* is a primary gene of the *qLTG-2* QTL.

## Discussion

### OsWRKY71 delays seed germination by positively upregulating important ABA signaling genes

Seed dormancy and germination are important agronomic traits. Internal factors block the germination of dormant seeds in the same environmental conditions (i.e., water, air, temperature) that are suitable for the germination of non-dormant seeds [64]. The switch from seed dormancy to germination has been studied extensively [65]; however, studies of the molecular mechanism underlying germinability or gemination speed remain scarce. This study demonstrates that the loss of *OsWRKY71* results in mutant seeds germinating earlier than wildtype (Fig. 3A, C). Early germination was observed in both water and on agar-solidified plant growth media (Figs. 3 and 5, and 7), suggesting that the phenotype is not oxic- or anoxic-dependent or affected by the presence of micro and macro plant nutrients. The *oswrky71* early germination phenotype also occurs in isolated embryos, suggesting contributions from the endosperm are not strictly required (Fig. 3B, D).

An RNA-seq time course analysis identified genes that were differentially expressed in dry and germinating *oswrky71* embryos. Notably, many genes involved in the ABA-dependent inhibition of seed germination were downregulated in *oswrky71*. Specifically, *oswrky71* showed reduced expression for multiple ABA-receptor components (*OsPYLs/RCARs*) that promote ABA signaling [43] and the *VP1-SDR4-DOG1L* branch of ABA signaling that promotes seed dormancy [45]. Early *oswrky71* seed germination was also hyposensitive to ABA treatments (Fig. 5). The RNA-seq data show that the OsWRKY71 transcription factor not only targets ABA signaling or hydrolytic enzyme genes, but is also involved in a wider web of signaling that includes other regulatory proteins. Figure 6 shows that other transcription factors that have been implicated in regulating germination, such as OsGATA3 and ERF20, are induced considerably earlier in *oswrky71-1*. With *OsGATA3*, the maximum mRNA level is four hours earlier and with *OsERF20*, it is eight hours earlier. Other examples include *OsVOZ1*, which has a consistently lower mRNA level than wildtype at all early germination time points we studied. Taken together, our data place OsWRKY71 upstream of other transcription factors in the signaling web that regulates germination and suggests that the transcription factor is a major negative regulator of germinations with both direct and indirect targets.

The perturbed hormone signaling characteristics of *oswrky71* mutants were not unexpected. Previously, OsWRKY71 was shown to negatively regulate the GA-dependent induction of an *HvAmy* gene in transiently transformed barley aleurone cells [28, 29]. This provided the expectation that rice mutants lacking OsWRKY71 would experience GA-hypersensitive increases in α-amylase activity in aleurone layers, which was confirmed here (Fig. 2). Furthermore, these GA-dependent increases in α-amylase activity were ABA hyposensitive in *oswrky71*, up to 0.05 µM. Thus, OsWRKY71 appears to be an important player in regulating the dynamic crosstalk between GA and ABA signals during rice seed germination.

**Fig. 8:**
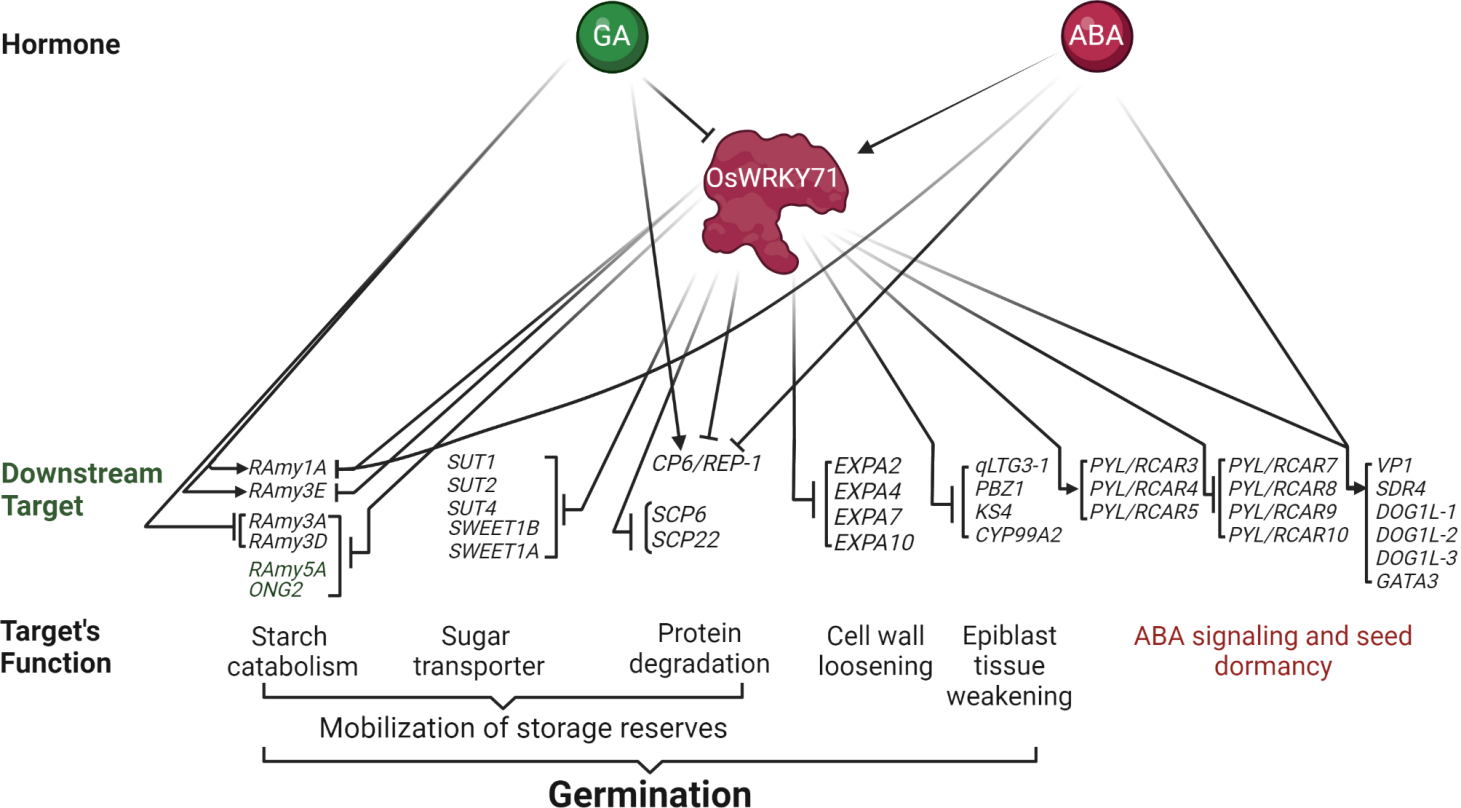
Model for OsWRKY71 as a negative regulator of rice seed germination. In this proposed model, OsWRKY71 acts as a transcriptional repressor of genes involved in the mobilization of storage reserves, including starch catabolism, sugar transport, and protein degradation. OsWRKY71 responds to the GA and ABA signals present during seed germination to modulate the expression of these target genes, either directly or indirectly. Besides storage-reserve mobilization, OsWRKY71 also appears to modulate the expression of genes involved in cell wall loosening and epiblast tissue weakening

It is interesting that expression of some ABA receptor genes was repressed while a few were enhanced (Figs. 5 and 8). PYL/RCAR family members exhibit functional diversity as ABA receptors. Some members are more involved in ABA signaling under specific developmental stages or environmental conditions, while others might have higher affinities for ABA. For example, *PYL/RCAR5* is a core component of ABA signaling in rice seeds [43]. Overall, ABA sensitivity could result from the combined effects of differential expression of these receptors. Specifically, the reduction in certain ABA receptor levels might weaken the signal transduction, while an increase in others may not fully compensate due to their distinct affinities or functional roles. Therefore, the altered expression of these receptors likely disrupts the balance required for optimal ABA signaling, leading to the observed hyposensitivity. It is intriguing to note that most of *PYL/RCAR* genes that are highly expressed in the embryo, namely *PYL/RCAR3*, *PYL/RCAR4*, and *PYL/RCAR5*, were downregulated. Further studies focusing on the specific roles and expression patterns of these *PYL/RCAR* members in *oswrky71* seeds could provide more insight into how this differential regulation contributes to the mutant phenotype.

### OsWRKY71 regulates seed reserve mobilization and cell wall remodeling genes

Many gene families showed changes in expression that could explain early germination in *oswrky71*. For example, genes encoding certain starch catabolism enzymes and sugar transporters were increased in *oswrky71* embryos (Supplemental Figure S5), which could increase rates of carbon mobilization and improve growth. Some of the most consistently upregulated genes in *oswrky71* encode glutelin SSPs, which are known to be metabolized during rice germination to feed the growing embryo [57]. The increases in glutelin expression also came with increases in SSP-metabolizing proteases and amino acid transporters (Supplemental Figure S5). Thus, *oswrky71* mutants could germinate early due to changes in gene expression that improve the mobilization of carbohydrate and protein seed storage reserves, as reported for other genes [66].

Genes involved in cell wall loosening and weakening were also altered in *oswrky71* in ways that could promote germination. For example, *EXPA* genes encoding cell wall loosening enzymes showed increased expression in dry *oswrky71* embryos, suggesting the *oswrky71* mutants might be primed for more rapid cell wall remodeling upon imbibition. Genes that weaken the epiblast, a protective covering of the embryo, were also increased in *oswrky71* at all time points. As the epiblast must be shed for germination to occur, there is potential that OsWRKY71 may delay seed germination by negatively regulating the expression of these epiblast-weakening genes.

Importantly, it is not currently possible to delineate which *oswrky71* gene expression changes occur due to (1) the loss of direct OsWRKY71 regulation, (2) the impacts of reduced ABA signaling components, or (3) those that result from being developmentally ahead of wildtype controls. Regardless of the mechanism underlying these changes, we have summarized this data into a working model that describes how OsWRKY71 coordinates hormone signals to negatively regulate seed germination (Fig. 8).

### OsWRKY71 regulates low-temperature responses in seeds and leaves

OsWRKY71 plays an important role in regulating rice responses to cold-stress conditions. For example, evidence provided in this study indicates that *OsWRKY71* is a core gene in the *qLTG-2* loci associated with improved low-temperature seed germinability (Fig. 7). Others have demonstrated that overexpressing *OsWRKY71* can improve cold-stress tolerance in vegetative tissues [67]. This provides an interesting prediction that *oswrky71* mutants might display deficient cold-stress responses in vegetative tissues. While this research was focused on the biology of seed germination, we do not rule out that *oswrky71* mutants might display other nuanced vegetative phenotypes, such as deficient responses to abiotic or biotic stress.

ABA plays a vital role in both promoting seed dormancy and cold-stress responses [68]. OsWRKY71 blocks seed germination at low temperatures but promotes cold-stress responses in vegetative tissues. This is consistent with a model whereby OsWRKY71 positively regulates ABA signaling in both tissues. A recent report shows that overexpression of an E2 ubiquitin-conjugating gene, *OsUBC12* (LOC_Os05g38550), owing to a transposon insertion in its promoter, increases low-temperature germinability in *japonica* rice [69].

### Group IIa WRKYs emerged with seed-bearing plants

From an evolutionary perspective, there are some interesting correlations between the evolution of transcription factors and new plant processes. Group IIa *WRKY* transcription factors emerged simultaneously as both seeds (and hence seed germination) and more complex defense responses (Supplemental Figure S1). It is, therefore, interesting that OsWRKY71 regulates starch metabolism and germination in seeds (Figs. 2 and 3) and that overexpression of OsWRKY71 in vegetative tissues results in constitutive activation of specific pathogen defense genes and enhanced resistance to the pathogenic bacterium *Xanthomonas oryzae* pv. *oryzae* (*Xoo*) [33]. This suggests that these newly emerging plant processes came to be regulated by pre-existing transcription factors, with at least some of these transcription factors (i.e., OsWRKY71) evolving to play multiple roles in regulating multiple traits.

## Conclusions

The data presented here provides insight into seed germination processes and also sheds new light on how developmental and inducible processes in plants may interact and share molecular components. Initially, it was believed that many regulatory components of seed germination are unique to germination and post-germination growth. Our data clarifies that developmental processes and inducible defense processes share components and potentially interact. One such shared component is the rice transcription factor OsWRKY71, a central signaling node in seed germination and responses to both biotic (disease) and abiotic (cold) stresses [33, 67]. Thus, OsWRKY71 warrants further research as it acts in multiple important signaling pathways and responds to different signals and plant hormones, including GA, ABA, ethylene, salicylic acid, and brassinosteroids. More importantly, future studies are needed to eliminate the possibility that differentially expressed genes in *oswrky71-1* merely result from being developmentally ahead of wild-type.

There are also critical agronomic benefits that can emerge from identifying early germinating rice mutants. For example, the rapid and uniform germination of seeds is beneficial to agricultural (e.g., crop breeding and management) and brewing industries (e.g., malting). Early germination at low temperatures is also essential for rice varieties adapted to direct seeding culture. We predict that *OsWRKY71* and orthologs in other angiosperms can be knocked out to produce early germinating seeds in various important seed crops (e.g., corn, wheat, sorghum, soybean, etc.).

### Experimental procedures

#### Evolution analysis

The procedure of evolution analysis is described in Method S1.

#### Plant materials and growth conditions

*Oryza sativa* ssp. japonica cv. Nipponbare *dSpm* mutants were acquired from the Sundaresan Lab, University of California, Davis [34]. Wildtype Nipponbare seeds were kindly provided by the Dale Bumpers National Rice Research Center - USDA ARS. Seeds were sterilized with 10% (v/v) bleach (Clorox) for 20 min and then washed 5 times with sterile Milli-Q water for 2 min per wash. Germination occurred in a dark incubator at 28 °C. After 14 days, seedlings were transferred to a 3:1 soil blend of Turface MVP calcined clay (Turface) and Lawn soil (Sta-Green) for growth in the greenhouse. Plants were flooded every 6 h with water supplemented with All Purpose Plant Food (Miracle-Gro). Growth conditions were maintained at 50–60% humidity, 28 ± 5 °C, with shaded natural lighting. Mutants were bred and verified to be homozygous by PCR, using oligonucleotides listed in Supplemental Table S2. Seed dormancy was broken by storing the seed dark and dry at room temperature for at least 1 month before germination assays.

### Germination assays

Wildtype and mutant seeds that were grown, harvested, and stored in parallel were sterilized as described above. Embryos were isolated from dry seeds as described by Siao et al. [70]. For a more detailed procedure, please see Methods S3. All germination assays occurred in a dark 28ºC incubator, using at least 25 seeds per replicate and at least three replicates. For liquid germination assays, 15 mL of sterile Milli-Q water was used in a 50 mm Petri dish. Solid germination assays occurred on ½x Murashige and Skoog Media (pH 5.8) supplemented with 1% (w/v) agar and 0.05% (w/v) MES. For ABA germination assays, these solid plates were supplemented with either solvent control (0.1% ethanol) or varying concentrations of ABA (GoldBio). Germination was determined by a radicle or coleoptile growth of at least 2 mm, an established method [36, 37]. Statistical significance in germination rate between wildtype and *oswrky71* mutants at each time point was determined via unpaired Student’s *t-tests*. T_50_ calculations were performed as previously described [71], and statistical significance was determined via Tukey’s Honestly Significant Difference test.

### Starch metabolism enzyme activity assays in embryo-less half seeds

Three replicates of 15 seeds were prepared by removing the GA-producing embryo and scutellum tissues and sterilizing them as described above. These prepared half seeds were placed on Whatman filter paper with a 10 mL incubation solution (20 mM succinic acid, 20 mM CaCl_2_, pH 5.8) for 48 h and then treated with or without 5 µM GA or 5 µM GA plus 2 µM ABA for 20 h. Amylase activity assays were performed using a modified method [72].

### RNA extractions and qRT-PCR

Method S3 documents the procedure of RNA isolation from embryos, cDNA synthesis and qRT-PCR.

### RNA-seq and data analysis

RNA sequencing was performed using the Illumina NextSeq 2000 system at the Nevada Genomics Center, which generated single-end 68 bp reads. Reads were trimmed to remove adapters and the final bp, which is of low quality due to the sequencing chemistry of Illumina NextSeq 2000. HISAT2 [73] was used to align reads to the rice genome (MSU Rice Genome Annotation Project Release 7; [74]. Uniquely mapped reads were counted using featureCounts [75]. Identification of DEGs was performed using DESeq2 (version 1.34.0) with the likelihood ratio test [76]. Genes with false discovery rate (FDR) adjusted p-values (or q-values) of 0.05 or less were considered as differentially expressed. Principal Component Analysis (PCA) was performed using plotPCA. Groups of DEGs with similar expression profiles were identified using DEGreport (version 1.30.0) and degPatterns [77].

### Bioinformatic analysis and graphics generation

For ab initio identification of cis-motifs, promoter sequences 2 kb upstream of the transcription start site for each gene were retrieved from the Rice Annotation Project Database (RAP-DB) [78]. DREME was used for ab initio analysis of cis-acting elements in the promoters of differentially expressed genes (https://meme-suite.org/meme/) (Bailey, 2011). Although it was initially designed for Chip-seq data analysis, this software can be used to identify enriched motifs from the cis-regulatory elements of differentially expressed genes identified by RNA-seq.

Method S4 describes GO analysis, and graphics generation. Method S5 documents the procedure for QTL analysis and an association study.

## Electronic supplementary material

Below is the link to the electronic supplementary material.

Supplementary Material 1

Supplementary Material 2

Supplementary Material 3

Supplementary Material 4

Supplementary Material 5

Supplementary Material 6

Supplementary Material 7

Supplementary Material 8

Supplementary Material 9

## Data Availability

The datasets generated during the current study are available in the NCBI SRA repository with the BioProject accession number PRJNA1155004.

## Abbreviations

ABA: Abscisic acid
ABF1: ABRE BINDING FACTOR 1
ABI3: ABSCISIC ACID INSENSITIVE 3
DEG: Differentially expressed genes
DOG1Ls: Delay of Germination 1-likes
ERF20: Ethylene Response Factor 20
GA: Gibberellins
GAMYB: GA-responsive MYB
GATA3: GATA transcription factor 3
GERMSP: Germination speed
HAI: Hours after imbibition
NCED5: NINE-CIS-EPOXYCAROTENOID DIOXYGENASE 5
PCA: Principal Component Analysis
PYL/RCARs: Pyrabactin resistance-like/Regulatory component of ABA receptors
qRT-PCR: Quantitative Reverse Transcription-PCR
SDR4: Seed Dormancy 4
VOZ1: Vascular plant One-Zinc finger 1
VP1: VIVIPAROUS-1
