# Supplementary material for: Disruption of the OsWRKY71 transcription factor gene results in early rice seed germination under normal and cold stress conditions
Source: BMC Plant Biol. 2024 Nov 18;24:1090. doi: 10.1186/s12870-024-05808-9 (PMC11571745; doi:10.1186/s12870-024-05808-9)
Supplement: Supplementary file 1 — Supplementary Material 1 [file 12870_2024_5808_MOESM1_ESM.pptx]

## Slide 1
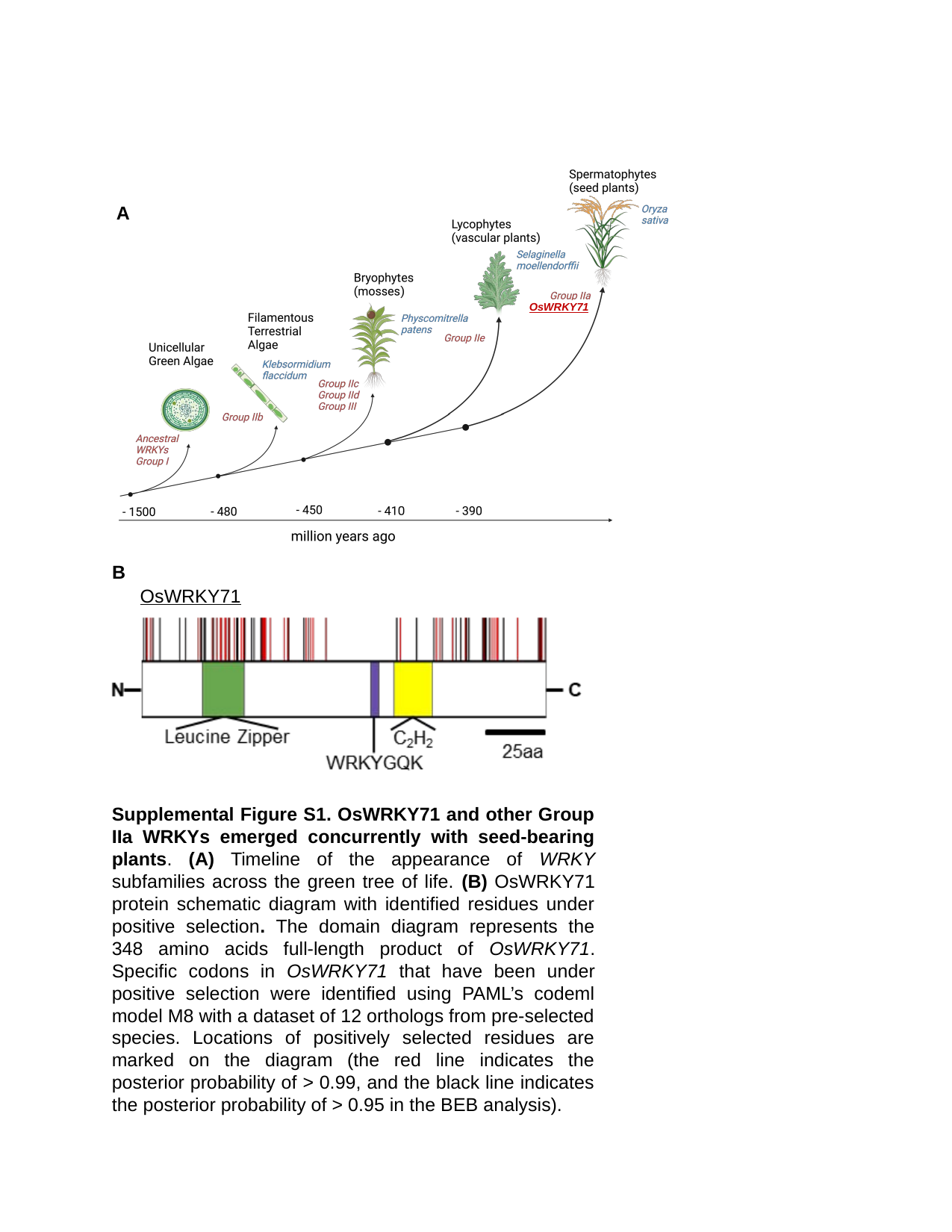

A
OsWRKY71
B
OsWRKY71
Supplemental Figure S1. OsWRKY71 and other Group IIa WRKYs emerged concurrently with seed-bearing plants. (A) Timeline of the appearance of WRKY subfamilies across the green tree of life. (B) OsWRKY71 protein schematic diagram with identified residues under positive selection. The domain diagram represents the 348 amino acids full-length product of OsWRKY71. Specific codons in OsWRKY71 that have been under positive selection were identified using PAML’s codeml model M8 with a dataset of 12 orthologs from pre-selected species. Locations of positively selected residues are marked on the diagram (the red line indicates the posterior probability of > 0.99, and the black line indicates the posterior probability of > 0.95 in the BEB analysis).

## Slide 2
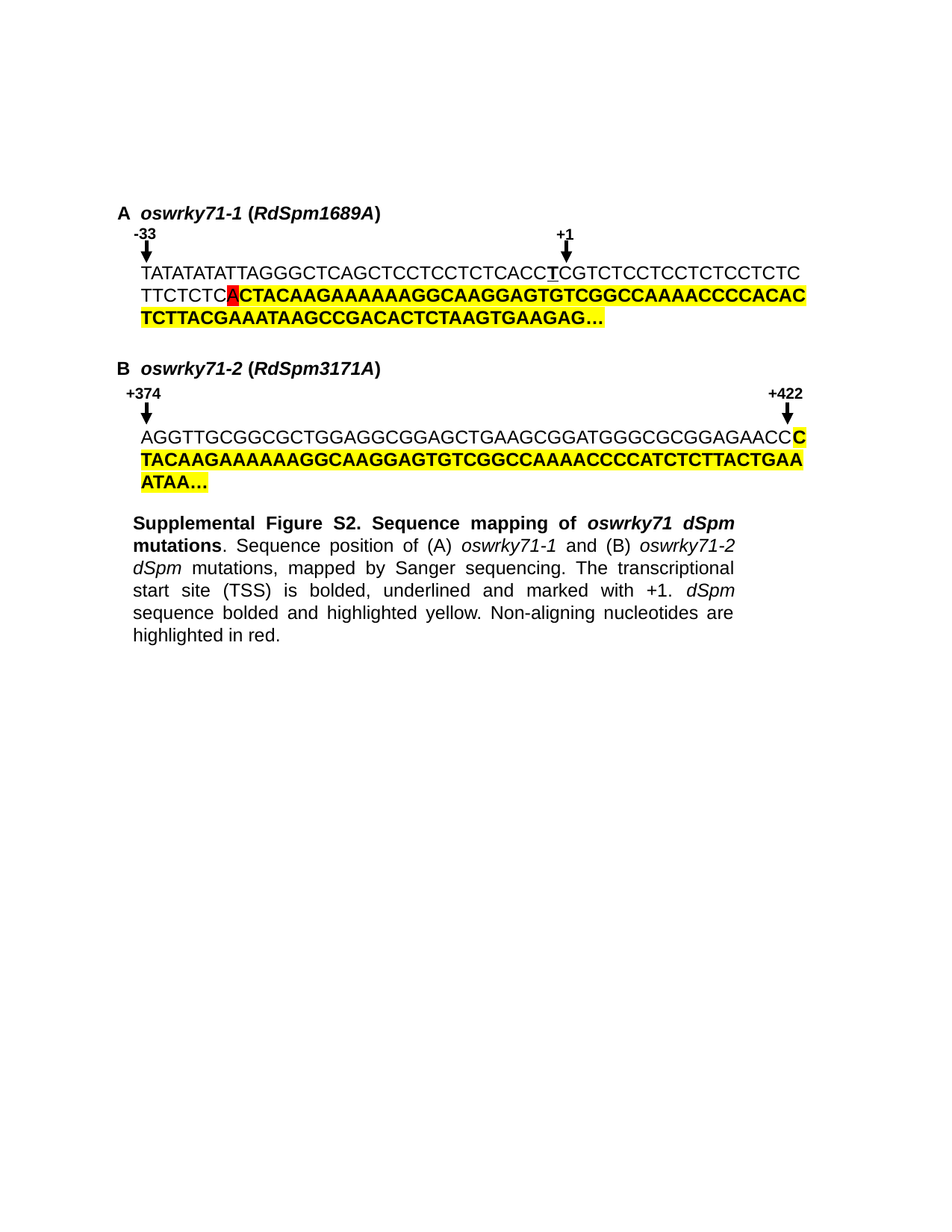

A oswrky71-1 (RdSpm1689A)
-33
+1
TATATATATTAGGGCTCAGCTCCTCCTCTCACCTCGTCTCCTCCTCTCCTCTCTTCTCTCACTACAAGAAAAAAGGCAAGGAGTGTCGGCCAAAACCCCACACTCTTACGAAATAAGCCGACACTCTAAGTGAAGAG…
B oswrky71-2 (RdSpm3171A)
+374
+422
AGGTTGCGGCGCTGGAGGCGGAGCTGAAGCGGATGGGCGCGGAGAACCCTACAAGAAAAAAGGCAAGGAGTGTCGGCCAAAACCCCATCTCTTACTGAAATAA…
Supplemental Figure S2. Sequence mapping of oswrky71 dSpm mutations. Sequence position of (A) oswrky71-1 and (B) oswrky71-2 dSpm mutations, mapped by Sanger sequencing. The transcriptional start site (TSS) is bolded, underlined and marked with +1. dSpm sequence bolded and highlighted yellow. Non-aligning nucleotides are highlighted in red.

## Slide 3
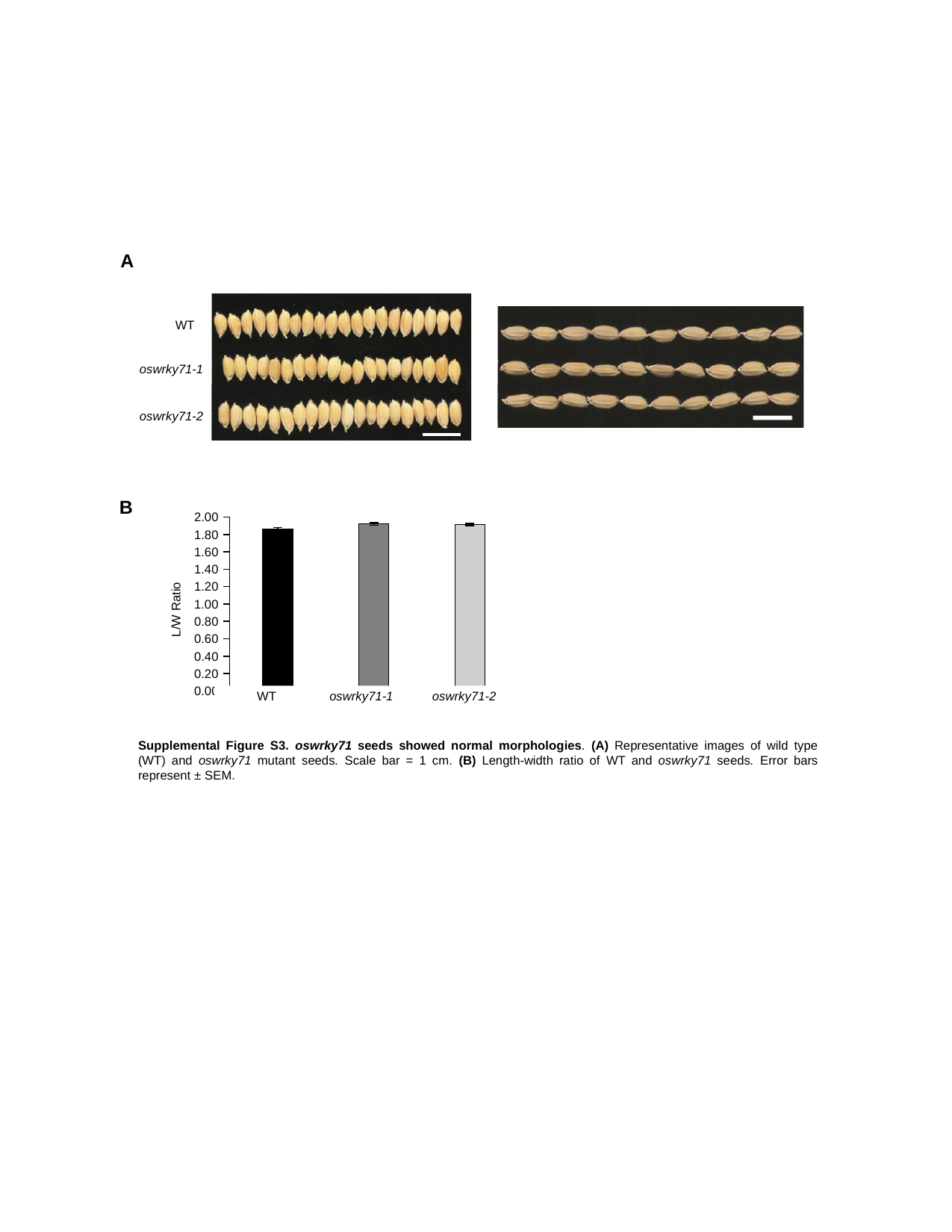

A
WT
oswrky71-1
oswrky71-2
B
### Chart
| Category | |
|---|---|
| WT | 1.8655563747508046 |
| wrky71-1 | 1.9211786295152502 |
| wrky71-2 | 1.9133275886479195 |WT oswrky71-1 oswrky71-2
Supplemental Figure S3. oswrky71 seeds showed normal morphologies. (A) Representative images of wild type (WT) and oswrky71 mutant seeds. Scale bar = 1 cm. (B) Length-width ratio of WT and oswrky71 seeds. Error bars represent ± SEM.

## Slide 4
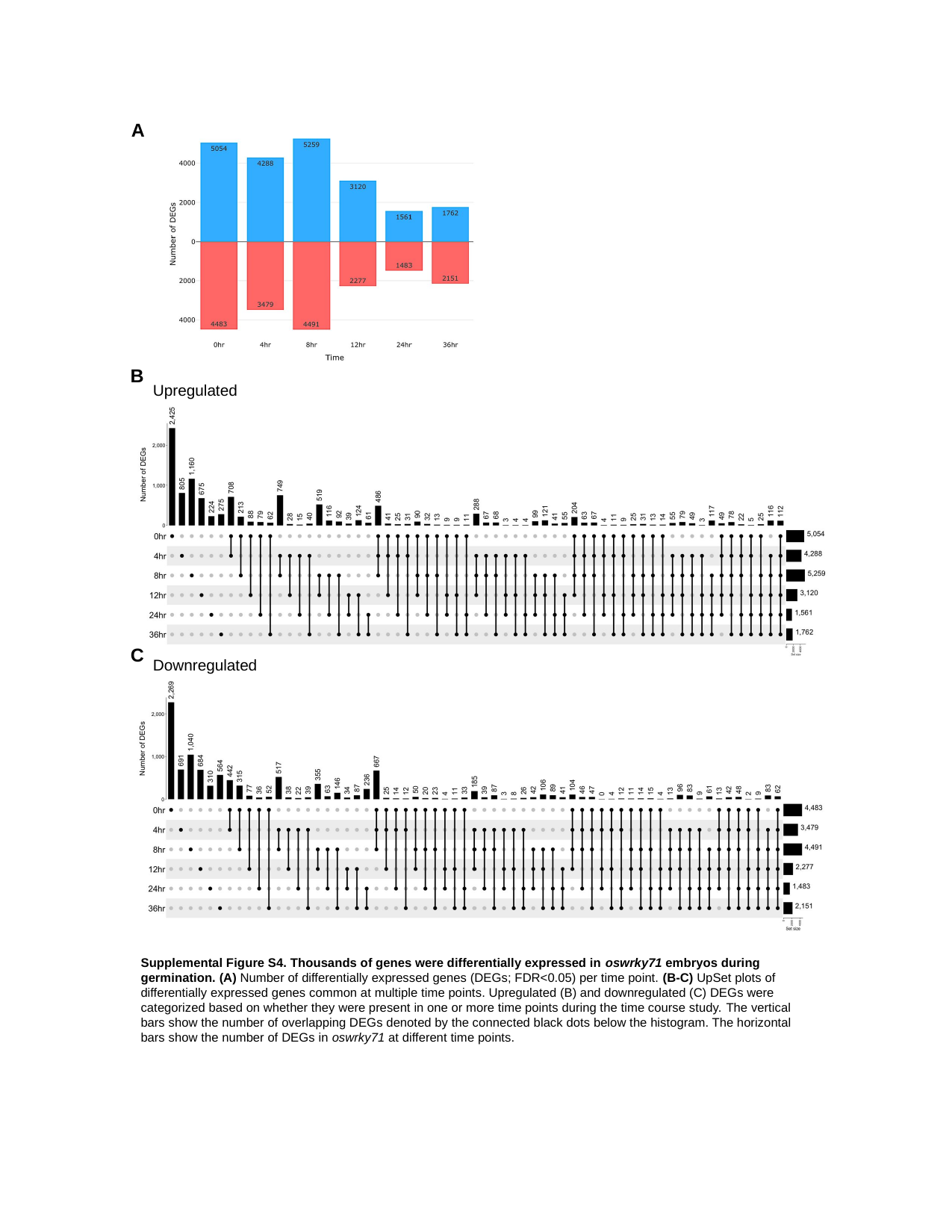

A
B
Upregulated
C
Downregulated
Supplemental Figure S4. Thousands of genes were differentially expressed in oswrky71 embryos during germination. (A) Number of differentially expressed genes (DEGs; FDR<0.05) per time point. (B-C) UpSet plots of differentially expressed genes common at multiple time points. Upregulated (B) and downregulated (C) DEGs were categorized based on whether they were present in one or more time points during the time course study. The vertical bars show the number of overlapping DEGs denoted by the connected black dots below the histogram. The horizontal bars show the number of DEGs in oswrky71 at different time points.

## Slide 5
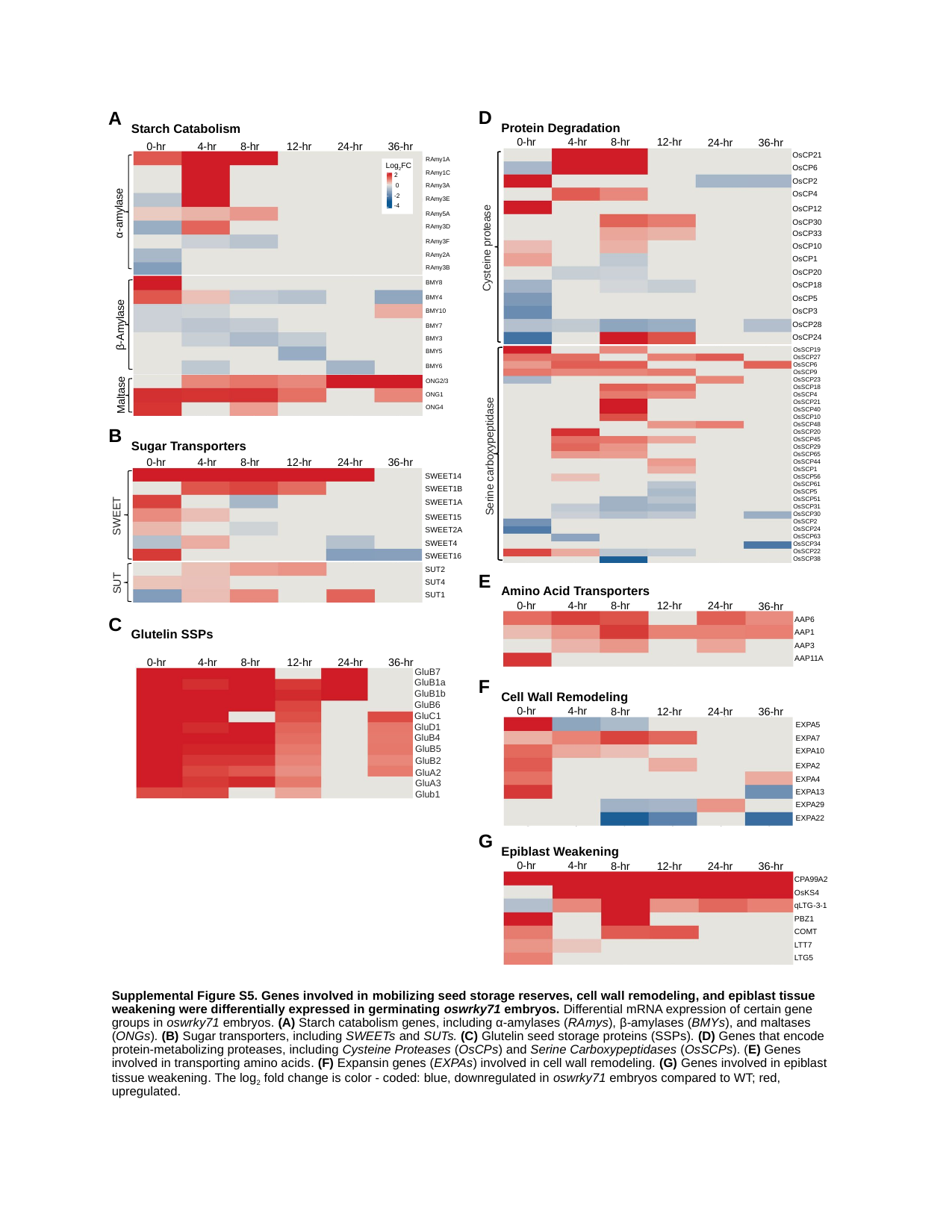

D
A
Protein Degradation
Starch Catabolism
0-hr
4-hr
8-hr
12-hr
24-hr
36-hr
0-hr
4-hr
8-hr
12-hr
24-hr
36-hr
OsCP21
OsCP6
OsCP2
OsCP4
OsCP12
OsCP30
OsCP33
OsCP10
OsCP1
OsCP20
OsCP18
OsCP5
OsCP3
OsCP28
OsCP24
RAmy1A
RAmy1C
RAmy3A
RAmy3E
RAmy5A
RAmy3D
RAmy3F
RAmy2A
RAmy3B
BMY8
BMY4
BMY10
BMY7
BMY3
BMY5
BMY6
ONG2/3
ONG1
ONG4
Log2FC
2
0
-2
-4
α-amylase
Cysteine protease
β-Amylase
OsSCP19
OsSCP27
OsSCP6
OsSCP9
OsSCP23
OsSCP18
OsSCP4
OsSCP21
OsSCP40
OsSCP10
OsSCP48
OsSCP20
OsSCP45
OsSCP29
OsSCP65
OsSCP44
OsSCP1
OsSCP56
OsSCP61
OsSCP5
OsSCP51
OsSCP31
OsSCP30
OsSCP2
OsSCP24
OsSCP63
OsSCP34
OsSCP22
OsSCP38
Maltase
B
Serine carboxypeptidase
Sugar Transporters
0-hr
4-hr
8-hr
12-hr
24-hr
36-hr
SWEET14
SWEET1B
SWEET1A
SWEET15
SWEET2A
SWEET4
SWEET16
SUT2
SUT4
SUT1
SWEET
E
SUT
Amino Acid Transporters
0-hr
4-hr
8-hr
12-hr
24-hr
36-hr
C
AAP6
AAP1
AAP3
AAP11A
Glutelin SSPs
0-hr
4-hr
8-hr
12-hr
24-hr
36-hr
GluB7
GluB1a
GluB1b
GluB6
GluC1
GluD1
GluB4
GluB5
GluB2
GluA2
GluA3
Glub1
F
Cell Wall Remodeling
0-hr
4-hr
8-hr
12-hr
24-hr
36-hr
EXPA5
EXPA7
EXPA10
EXPA2
EXPA4
EXPA13
EXPA29
EXPA22
G
Epiblast Weakening
0-hr
4-hr
8-hr
12-hr
24-hr
36-hr
CPA99A2
OsKS4
qLTG-3-1
PBZ1
COMT
LTT7
LTG5
Supplemental Figure S5. Genes involved in mobilizing seed storage reserves, cell wall remodeling, and epiblast tissue weakening were differentially expressed in germinating oswrky71 embryos. Differential mRNA expression of certain gene groups in oswrky71 embryos. (A) Starch catabolism genes, including α-amylases (RAmys), β-amylases (BMYs), and maltases (ONGs). (B) Sugar transporters, including SWEETs and SUTs. (C) Glutelin seed storage proteins (SSPs). (D) Genes that encode protein-metabolizing proteases, including Cysteine Proteases (OsCPs) and Serine Carboxypeptidases (OsSCPs). (E) Genes involved in transporting amino acids. (F) Expansin genes (EXPAs) involved in cell wall remodeling. (G) Genes involved in epiblast tissue weakening. The log2 fold change is color - coded: blue, downregulated in oswrky71 embryos compared to WT; red, upregulated.

## Slide 6
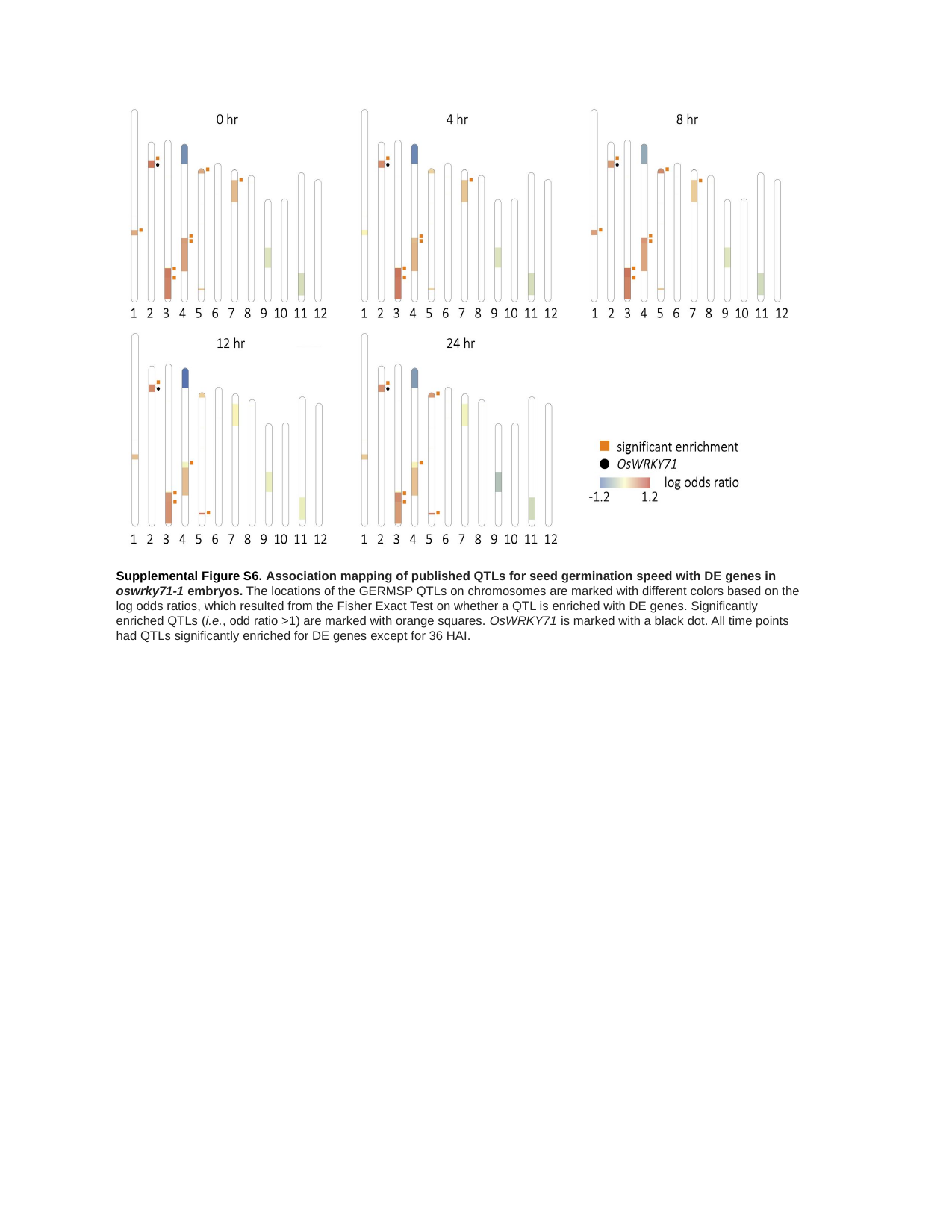

Supplemental Figure S6. Association mapping of published QTLs for seed germination speed with DE genes in oswrky71-1 embryos. The locations of the GERMSP QTLs on chromosomes are marked with different colors based on the log odds ratios, which resulted from the Fisher Exact Test on whether a QTL is enriched with DE genes. Significantly enriched QTLs (i.e., odd ratio >1) are marked with orange squares. OsWRKY71 is marked with a black dot. All time points had QTLs significantly enriched for DE genes except for 36 HAI.
