# Supplementary material for: Disruption of the OsWRKY71 transcription factor gene results in early rice seed germination under normal and cold stress conditions
Source: BMC Plant Biol. 2024 Nov 18;24:1090. doi: 10.1186/s12870-024-05808-9 (PMC11571745; doi:10.1186/s12870-024-05808-9)
Supplement: Supplementary file 2 — Supplementary Material 2 [file 12870_2024_5808_MOESM2_ESM.docx]

**Supplemental methods and references**

**Methods S1.** **Evolution analysis**

The amino acid sequences of the complete WRKY gene families from representative species across the tree of life (*Arabidopsis thaliana, Glycine max, Brachypodium distachyon, Selaginella moellendorffii, Physcomitrella patens, Chlamydomonas reinhardtii, Chlorella variabilis, Coccomyxa subellipsoidea, Micromonas pusilla, Ostreococcus lucimarinus, Ostreococcus tauri, Volvox carteri, Klebsormidium flaccidum, Bathycoccus prasinos, Dictyostelium discoideum, Polysphondylium pallidum, Dictyostelium fasciculatum, Fonticula alba, Acanthamoeba castellanii, Giardia lamblia, Giardia intestinalis, Dictyostelium purpureum, Auxenochlorella protothecoides, Spironucleus salmonicida, Mucor circinelloides, Rhizopus delemar, Absidia idahoensis, Lichtheimia corymbifera, Rhizophagus irregularis*, and *Mortierella verticillate*) were determined by searching phytozome (<http://www.phytozome.net/>) or NCBI (<http://www.ncbi.nlm.nih.gov/>). Amino acid sequences of a representative member of the WRKY domain from each of the WRKY gene subfamilies (Groups I, IIa, IIb, IIc, IId, IIe, and III) was used for the searches and amino acid comparisons and phylogenetic analyses were performed on all discovered WRKY genes as described (Rinerson *et al.*, 2015) to determine the presence or absence of each subfamily in the species. The presence of the conserved PR and VQR introns was also used to verify the subfamily of WRKY genes in each species (Rinerson *et al.*, 2015).

Using the full-length OsWRKY71 amino acid sequence as a query for BLASTp, 12 ortholog sequences of representative species were collected from the Plaza 4.0 protein database (<https://bioinformatics.psb.ugent.be/plaza>) (Van Bel *et al.*, 2018), except for *Micromonas pusilla* which was collected from (<https://mycocosm.jgi.doe.gov>)(Grigoriev *et al.*, 2014) and fern species *Salvinia cucullata* and *Azolla filiculoides* which were downloaded from (<https://marchantia.info/>) (Montgomery *et al.*, 2020). List of species and orthologs used in the dataset are in Supplemental Table S1A.

Full protein sequences of orthologs were filtered for confidence using the GUIDANCE2 server (Sela *et al.*, 2015) with the MAFFT MSA algorithm and 100 bootstrap iterations. Residues with scores less than 90% were filtered out, leaving 87.8% of residues in the alignment. Filtered sequences were back-translated into coding domain sequences with EMBOSS Backtranseq (Madeira *et al.*, 2019) using the codon usage table for corresponding species. PAL2NAL(Suyama *et al.*, 2006) was used to convert protein multiple sequence alignments and corresponding back-translated CDS into codon alignments using the Universal Codon Table. jModelTest 2 (Darriba *et al.*, 2012) was used to derive the nucleotide substitution model used for building maximum likelihood trees, and tree files were used as input files for PAML.

To detect positive selection on WRKY71, CodeML under the PAML v4.9 package (Yang, 2007) was performed using site models, which allowed for the ω (ratio of nonsynonymous/ synonymous substitution rates) to vary among codons in the protein sequence used. ω ratio is a measure of natural selection acting on a protein, and performing likelihood ratio tests of positive selection for two pairs of models in the program allows us to not only detect selection but also determine sites of high positive selection. Supplemental Table S1B summarizes the results of the analysis. To accurately identify sites that evolved under positive selection in the *OsWRKY71* gene, the Bayes Empirical Bayes approach was used for model M8 (Supplemental Table S1B).

**Methods S2. Imaging and** **time-lapse video recording**

All images were acquired using a MU1803 18 MP microscope camera (AmScope), utilizing AmScope software version 4.11. Germination video was captured using the time-lapse function set to image every 2 min.

**Methods S3. Embryo isolation, qRT-PCR and RNA-seq**

Rice grains were threshed using a compact rice husker (TR-200, Kett, Tokyo, Japan). Sterile nail clippers were used to remove >90% of the endosperm from the embryo. Then, a sterile a #11 X-Acto precision knife was used to chip, chisel, or peel away the remaining endosperm tissue, leaving an unwounded isolated embryo. Isolated embryos were sterilized with 10% (v/v) bleach (Clorox) for 1 minute (to account for the increased sensitivity of an exposed isolated embryo) and were then washed 5 times with sterile Milli-Q water, 2 minutes per wash.

Sterilized embryos were incubated in 2 mL of sterile Milli-Q water at 28ºC for the duration of each time point. Each sample contained 18 isolated embryos and three replicates were performed for each time point. After incubation, water was removed, and the samples were frozen in N_2_ (l) and stored at -80ºC. Embryos were pre-homogenized in N_2_ (l) using a mortar and pestle (Coors) and 1 mL of TRIzol (Thermo Fisher Scientific) was added to the frozen tissue. Each sample was fully homogenized using a 5 mL syringe and 20-gauge needle (B-D). Plant tissue and cell debris were pelleted by centrifugation at 12,000 xg for 10 minutes at 4ºC. The supernatant was transferred to a fresh tube and chloroform was added and mixed. Phases were separated by centrifugation at 10,000 xg for 15 minutes at 4ºC and the upper layer was transferred to a fresh tube. An equal volume of high-salt precipitation buffer (1.2 M NaCl, 0.8 M Na-citrate) and isopropanol was added to each sample and mixed. RNA was pelleted by centrifugation at 10,000 xg for 10 minutes at 4ºC. The supernatant was decanted, and RNA pellets were washed with 75% ethanol, followed by centrifugation at 10,000 xg for 10 minutes at 4ºC. The supernatant was decanted, and the RNA pellets were dried under a laminar flow hood (Forma Scientific). To remove any genomic DNA, samples were resuspended in RNase-free water and treated with a DNase Max Kit (Qiagen). RNA samples were then purified by a phenol:chloroform:isoamyl alcohol (25:24:1, pH = 4.7; Fisher Scientific) extraction, followed by a final clean-up using RNeasy Plant Mini Kits (Qiagen). RNA purity for all samples was determined using a Nanodrop ND-1000 (Thermo Fisher Scientific) and all samples had OD260/280 and OD260/230 values of >1.8 and >2.0, respectively.

For qRT-PCR, cDNA was synthesized using the Omniscript RT Kit (Qiagen). Primers targeting the 5’UTR/first exon and the 3’UTR of *OsWRKY71* were used (Supplemental Table S1). qRT-PCR was performed using QuantiTect SYBR Green PCR kit (Qiagen) and reactions were run on the CFX96 Touch Real-Time PCR Detection System (Bio-Rad). Reactions were run as follows: 95°C for 15 minutes; 40 cycles of 95°C for 15 seconds, 56°C for 30 seconds, and 72°C for 30 seconds. The expression of *OsWRKY71* was normalized to *Actin* and the 2^-ΔΔCt^ method (Livak and Schmittgen, 2001) was used to calculate the fold-change expression of *OsWRKY71* between wild type and *oswrky71* mutants.

The procedure for RNA-seq is documented in the Experimental Procedures section of the main body of the paper.

**Methods S4. *de novo* identification of *cis-*motifs, computation analysis and** **graphics generation**

Gene Ontology (GO) enrichment analysis was conducted using the package clusterProfiler (version 4.2.0) (Yu et al., 2012). The enricher function was used for an overrepresentation analysis using the hypergeometric distribution, the Bonferroni-Yekutieli (FDR under dependency) method for p-value adjustment, and a p-value cutoff of 0.05. The GO ID to gene name database was used from agriGO v2.0 (Tian et al., 2017). The GO ID to GO name database was used from the basic version of the GO from the Gene Ontology Consortium (Botstein et al., 2000). To visualize the results from the enrichment analysis, a heatmap showing the fold enrichment of the top 20 GO terms with the largest magnitude of enrichment across six time points was used. The heatmap was generated using the package ComplexHeatmap (version 2.10.0) (Gu et al.). The function "Heatmap" was used to both perform hierarchical clustering and visualize the heatmap. UpSet plots for DEG data sets were generated using ComplexHeatmap’s (version 2.10.0) UpSet function (Gu et al., 2016). Mirrored bar plots were generated using plotly.

**Method S5. Identification of early-expressed genes in germinating *oswrky71* embryos**

A cross lag correlation analysis was performed on the RNA-seq data by computing the cross-correlations between the gene ratios for all DEGs, using lags of 4-hour increments. This identified 103 candidate genes that potentially show early expression patterns in germinating *oswrky71* embryos. Separately, DESeq2 was used to identify genes that were differentially expressed in WT between each adjacent time point (*i.e.*, 0 vs 4 HAI, 4 vs 8 HAI, etc.), and this was repeated for *oswrky71*. From this we identified thousands of candidate genes that were DE in *oswrky71* mutants one time point earlier than WT, including 707 genes that encode transcription factors. Three candidates with exemplary early expression profiles were selected from this list and expression plots were generated in Microsoft excel.

**Method S6. Analysis of QTLs enriched with differentially expressed genes**

The QTL dataset (version 39), manually curated and quality controlled by Gramene (Ni *et al.*, 2009), was downloaded from the Gramene database. The original dataset includes 8,216 entries. QTLs sharing the same chromosome number, QTL start coordinate, QTL end coordinate, and trait name were considered duplicates and hence removed, resulting in 5,165 unique entries. If the coordinates of a gene model overlap with those of a QTL, the gene was considered to be associated with that QTL. Those genes, along with their expression data in each transcriptome, were retrieved using in-house PERL and R scripts.

Our focus was on the GERMSP category of QTLs, which contains 26 non-redundant entries, 22 of which are mapped to the rice genome. Only 20 of these entries contained an MSU7 gene model. To analyze the enrichment of DE genes in each QTL of the GERMSP category, the numbers of DE and nonDE genes of a given QTL were compared against those in the noQTL category, which includes all the genes that are not located in the GERMSP QTLs of the two categories at a given time point. Fisher’s Exact Test was used to test that the odd ratio is greater than 1 in R. The enrichment data were plotted RIdeogram (Hao *et al.*, 2020).

**References**

**Darriba, D., Taboada, G.L., Doallo, R. and Posada, D.** (2012) jModelTest 2: more models, new heuristics and parallel computing. *Nat. Methods*, **9**, 772-772.

**Grigoriev, I.V., Nikitin, R., Haridas, S., Kuo, A., Ohm, R., Otillar, R., Riley, R., Salamov, A., Zhao, X. and Korzeniewski, F.** (2014) MycoCosm portal: gearing up for 1000 fungal genomes. *Nucleic Acids Res.*, **42**, D699-D704.

**Hao, Z., Lv, D., Ge, Y., Shi, J., Weijers, D., Yu, G. and Chen, J.** (2020) RIdeogram: drawing SVG graphics to visualize and map genome-wide data on the idiograms. *PeerJ. Comput. Sci.*, **6**, e251.

**Livak, K.J. and Schmittgen, T.D.** (2001) Analysis of relative gene expression data using real-time quantitative PCR and the 2− ΔΔCT method. *Methods*, **25**, 402-408.

**Madeira, F., Park, Y.M., Lee, J., Buso, N., Gur, T., Madhusoodanan, N., Basutkar, P., Tivey, A.R., Potter, S.C. and Finn, R.D.** (2019) The EMBL-EBI search and sequence analysis tools APIs in 2019. *Nucleic Acids Res.*, **47**, W636-W641.

**Montgomery, S.A., Tanizawa, Y., Galik, B., Wang, N., Ito, T., Mochizuki, T., Akimcheva, S., Bowman, J.L., Cognat, V. and Maréchal-Drouard, L.** (2020) Chromatin organization in early land plants reveals an ancestral association between H3K27me3, transposons, and constitutive heterochromatin. *Curr. Biol.*, **30**, 573-588. e577.

**Ni, J., Pujar, A., Youens-Clark, K., Yap, I., Jaiswal, P., Tecle, I., Tung, C.-W., Ren, L., Spooner, W. and Wei, X.** (2009) Gramene QTL database: development, content and applications. *Database*, **2009**, bap005.

**Rinerson, C.I., Rabara, R.C., Tripathi, P., Shen, Q.J. and Rushton, P.J.** (2015) The evolution of WRKY transcription factors. *BMC Plant Biol.*, **15**, 1-18.

**Sela, I., Ashkenazy, H., Katoh, K. and Pupko, T.** (2015) GUIDANCE2: accurate detection of unreliable alignment regions accounting for the uncertainty of multiple parameters. *Nucleic Acids Res.*, **43**, W7-W14.

**Suyama, M., Torrents, D. and Bork, P.** (2006) PAL2NAL: robust conversion of protein sequence alignments into the corresponding codon alignments. *Nucleic Acids Res.*, **34**, W609-W612.

**Van Bel, M., Diels, T., Vancaester, E., Kreft, L., Botzki, A., Van de Peer, Y., Coppens, F. and Vandepoele, K.** (2018) PLAZA 4.0: an integrative resource for functional, evolutionary and comparative plant genomics. *Nucleic Acids Res.*, **46**, D1190-D1196.
