# Supplementary material for: Disruption of the OsWRKY71 transcription factor gene results in early rice seed germination under normal and cold stress conditions
Source: BMC Plant Biol. 2024 Nov 18;24:1090. doi: 10.1186/s12870-024-05808-9 (PMC11571745; doi:10.1186/s12870-024-05808-9)
Supplement: Supplementary file 4 — Supplementary Material 4 [file 12870_2024_5808_MOESM4_ESM.docx]

**Supplemental Movie Legend**

**Supplemental Movie S1.** Germinating wildtype, *oswrky71-1*, and *oswrky71-2* (left to right). Germination video was captured using the time-lapse function set to image every 2 min for a duration of 72 hours using MU1803 18 MP microscope camera (AmScope), utilizing AmScope software version 4.11.
