# Supplementary material for: Disruption of the OsWRKY71 transcription factor gene results in early rice seed germination under normal and cold stress conditions
Source: BMC Plant Biol. 2024 Nov 18;24:1090. doi: 10.1186/s12870-024-05808-9 (PMC11571745; doi:10.1186/s12870-024-05808-9)
Supplement: Supplementary file 5 — Supplementary Material 5 [file 12870_2024_5808_MOESM5_ESM.docx]

| **Supplemental Table S1A. *OsWRKY71* ortholog genes used in the evolutionary analysis.** | | |
| --- | --- | --- |
| **Species** | **Common Name** | **Gene ID** |
| *Oryza sativa* | Rice | *LOC_Os02g08440* |
| *Picea abies* | Norway Spruce | *MA_934202g0010* |
| *Ginkgo biloba* | Ginkgo | *GBI00002386* |
| *Arabidopsis thaliana* | Thale cress | *AT1G80840* |
| *Pinus taeda* | Loblolly pine | *PTA00063201* |
| *Marchantia polymorpha* subsp. *ruderalis* | Liverwort | *Mapoly0162s0003* |
| *Chlamydomonas reinhardtii* | Single-cell green algae | *Cre04.g228400* |
| *Selaginellae moellendorfii* | Spikemoss | *gene31554* |
| *Micromonas pusilla* | Green algae | *MicpuC3v2\|1336* |
| *Glycine max* | Soybean | *Glyma.08G218600* |
| *Amborella trichopoda* | Amborella | *ATR0739G003* |
| *Salvinia cucullata* | Floating fern | *Sacu_v1.1_s0216.g026051* |
| *Azolla filiculoides* | Water fern | *Azfi_s0019.g015016* |

| **Supplemental Table S1B. Parameter estimates and likelihood scores for the evolution analysis of *OsWRKY71* and its orthologs.** | | | | | | |  |
| --- | --- | --- | --- | --- | --- | --- | --- |
| Model* | dN/dS** | Parameter estimates*** | PSS**** | lnL | 2∆l | *P*-value | |
| M0: one-ratio (1) | 0.49 | ω =0.49 | N.A. | -11873 |  |  | |
| M3: discrete (5) |  |  |  |  |  |  | |
| M1a: neutral (1) | 0.74 | p0=0.30  , (p1=0.71) | not allowed | -11613 |  |  | |
|  |  | (ω0=0.11), (ω1=1.00) |  |  |  |  | |
| M2a: selection (3) | 2.15 | p0=0.22, p1=0.46, (p2=0.32) | 27(7) | -11555 |  |  | |
|  |  | (ω0=0.10), (ω1=1), ω2=5.19 |  |  |  |  | |
| M1a vs M2a |  |  |  |  | 114.67 | p<0.001 | |
|  |  |  |  |  |  |  | |
| M7: beta | 0.63 | p=0.44, q=0.25 | not allowed | -11592 |  |  | |
| M8: beta & ω | 1.77 | p0=0.68, (p1=0.32) | 171(57) | -11542 |  |  | |
|  |  | p=0.43, q=0.28, ω=4.31 |  |  |  |  | |
| M7 vs M8 |  |  |  |  | 99.55 | p<0.001 | |

*Number in parenthesis is the number of free parameters in the ω distribution

**This dN/dS ratio is an average over all sites in the gene alignment

***Parameters in parentheses are not free parameters

****PSS=number of positive selection sites based on BEB Analysis. First number is the PSS with posterior probabilities > 50%, numbers in parenthesis has probabilities > 95%

Assuming a single ω ratio across all sites, 0.49 was the estimated general ω value.

This is expected, as ω ratio over all sites of a protein is almost never > 1. To account for positive selection, we tested codon models M2a and M8 against M1a and M7, respectively. The M2a model adds a third class of sites with ω > 1 (selection) while M1a maintains a neutral model with two classes ω < 1 and ω = 1. Testing our data set for M2a against M1a shows that there was a significantly better fit for the data when selection was allowed (LRT statistic: 114.67, p-value < 0.001). This suggests that some sites of the WRKY71 have indeed undergone adaptive evolution. Model M8 on the one hand, allows for an extra class of sites with ω > 1 in addition to model M7’s ten classes with ω < 1 in a flexible beta-distribution. Our comparison of M8 vs M7 yet again reveals a better fit of the data for the M8 model with positive selection (LRT statistic: 99.55, p-value < 0.001).

**Supplemental Table S2. Oligonucleotides for genotyping and qRT-PCR.**

Primer ID Sequence (5′ to 3) Purpose Target region

10042 GAGATCTTGGGCTTGCACTC Genotyping *OsWRKY71*

10043 CGTCTTATCCACTCAAAAACCC Genotyping *OsWRKY71*

10044 CTTCTTCTTGACAGGGCAGG Genotyping *OsWRKY71*

10045 GGAGCAGCAGAAAAGTTTGC Genotyping *OsWRKY71*

10050 GAGCGTCCATTTTAGAGTGAC Genotyping *dSpm*

10009 AGCAAGAACACGATCGACGC qRT-PCR *OsWRKY71*

10010 GATCGTTGGTTGATCAGCCATG qRT-PCR *OsWRKY71*

18011 TGGCATCTCTCAGCACATTCC qRT-PCR Actin (control)

18012 TGCACAATGGATGGGCCAGA qRT-PCR Actin (control)

The locus numbers for the OsWRKY71 and Actin genes are LOC_Os02G08440 and LOC_Os03g50885, respectively.

**Supplemental Table S3. Read counts and mapping frequency of RNA-seq data.**

|  | | | | | |
| --- | --- | --- | --- | --- | --- |
| **Sample** | **Total Reads** | **Uniquely Mapped (Reads)** | **Uniquely Mapped (%)** | **Multi Mapped (Reads)** | **Total % Mapped** |
| Wildtype 0 HAI-1 | 90,862,863 | 38,600,805 | 42.48 | 48,152,514 | 95.48 |
| Wildtype 0 HAI-2 | 99,745,547 | 41,092,854 | 41.20 | 54,002,765 | 95.34 |
| Wildtype 0 HAI-3 | 86,493,098 | 36,787,537 | 42.53 | 45,664,794 | 95.33 |
| Wildtype 4 HAI-1 | 74,514,092 | 43,077,760 | 57.81 | 27,118,027 | 94.20 |
| Wildtype 4 HAI-2 | 87,238,800 | 48,570,518 | 55.68 | 34,083,503 | 94.74 |
| Wildtype 4 HAI-3 | 88,293,981 | 47,951,969 | 54.31 | 35,726,024 | 94.77 |
| Wildtype 8 HAI-1 | 72,813,370 | 46,906,528 | 64.42 | 21,081,469 | 93.37 |
| Wildtype 8 HAI-2 | 81,254,557 | 45,433,749 | 55.92 | 31,184,804 | 94.29 |
| Wildtype 8 HAI-3 | 57,675,074 | 39,489,894 | 68.47 | 14,250,295 | 93.18 |
| Wildtype 12 HAI-1 | 59,584,287 | 43,787,125 | 73.49 | 11,313,453 | 92.48 |
| Wildtype 12 HAI-2 | 63,643,000 | 44,838,490 | 70.45 | 14,053,544 | 92.53 |
| Wildtype 12 HAI-3 | 63,468,061 | 47,490,739 | 74.83 | 11,265,745 | 92.58 |
| Wildtype 24 HAI-1 | 63,326,291 | 47,851,178 | 75.56 | 10,664,249 | 92.40 |
| Wildtype 24 HAI-2 | 66,772,030 | 51,022,892 | 76.41 | 10,713,390 | 92.46 |
| Wildtype 24 HAI-3 | 63,710,231 | 48,471,404 | 76.08 | 10,474,091 | 92.52 |
| Wildtype 36 HAI-1r | 80,012,892 | 60,074,977 | 75.08 | 14,052,975 | 92.65 |
| Wildtype 36 HAI-2r | 62,662,970 | 57,761,036 | 90.20 | 4,901,934 | 97.30 |
| Wildtype 36 HAI-3 | 67,321,959 | 52,287,558 | 77.67 | 10,085,425 | 92.65 |
| *oswrky71-1* 0 HAI-1 | 84,555,973 | 40,247,420 | 47.60 | 39,971,952 | 94.87 |
| *oswrky71-1* 0 HAI-2 | 89,492,905 | 45,661,517 | 51.02 | 39,182,394 | 94.81 |
| *oswrky71-1* 0 HAI-3 | 74,118,279 | 41,890,467 | 56.52 | 28,016,726 | 94.32 |
| *oswrky71-1* 4 HAI-1 | 68,829,413 | 42,652,668 | 61.97 | 22,199,303 | 94.22 |
| *oswrky71-1* 4 HAI-2 | 60,765,836 | 40,335,650 | 66.38 | 16,584,030 | 93.67 |
| *oswrky71-1* 4 HAI-3 | 64,009,135 | 41,665,784 | 65.09 | 18,281,986 | 93.66 |
| *oswrky71-1* 8 HAI-1 | 64,114,909 | 47,234,753 | 73.67 | 12,219,094 | 92.73 |
| *oswrky71-1* 8 HAI-2 | 70,702,826 | 53,701,223 | 75.95 | 11,716,710 | 92.53 |
| *oswrky71-1* 8 HAI-3 | 62,601,630 | 46,554,030 | 74.37 | 11,458,368 | 92.67 |
| *oswrky71-1* 12 HAI-1 | 59,463,015 | 44,435,659 | 74.73 | 10,597,117 | 92.55 |
| *oswrky71-1* 12 HAI-2 | 60,587,202 | 46,328,444 | 76.47 | 9,501,890 | 92.15 |
| *oswrky71-1* 12 HAI-3 | 60,516,685 | 47,457,352 | 78.42 | 8,462,893 | 92.40 |
| *oswrky71-1* 24 HAI-1 | 64,614,070 | 46,882,827 | 72.56 | 13,092,816 | 92.82 |
| *oswrky71-1* 24 HAI-2 | 57,173,374 | 44,527,710 | 77.88 | 8,264,588 | 92.34 |
| *oswrky71-1* 24 HAI-4r | 56,437,024 | 41,369,747 | 73.30 | 10,980,871 | 92.76 |
| *oswrky71-1* 36 HAI-1 | 125,937,532 | 83,928,126 | 66.64 | 33,925,768 | 93.58 |
| *oswrky71-1* 36 HAI-2r | 86,545,587 | 65,326,129 | 75.48 | 15,126,512 | 92.96 |
| *oswrky71-1* 36 HAI-3r | 88,581,423 | 66,881,892 | 75.50 | 15,191,375 | 92.65 |

The output metrics for RNA-seq samples are listed, including the total number of reads, uniquely mapped reads, % of reads uniquely mapped, number of multi-mapped reads, and total % of reads mapped.
